# Dynamic Modulation of OECT‐Based Inverters for In Situ Electrophysiological Monitoring

**DOI:** 10.1002/advs.202512755

**Published:** 2025-10-17

**Authors:** Guohong Hu, Qijun Cai, Zhenglei Liu, Rongsheng Zeng, Liang‐Wen Feng, Jianhua Chen, Shiji Xiahou, Wei Huang

**Affiliations:** ^1^ School of Automation Engineering University of Electronic Science and Technology of China (UESTC) No.2006, Xiyuan Ave, West Hi‐Tech Zone Chengdu Sichuan 611731 China; ^2^ Key Laboratory of Green Chemistry & Technology Ministry of Education College of Chemistry Sichuan University 24 South Section 1, 1st Ring Road Chengdu Sichuan 610065 China; ^3^ Department of Chemical Science and Technology Yunnan University South Section, East Outer Ring Road, Chenggong New District Kunming Yunnan 650504 China

**Keywords:** dynamic gain modulating system, in situ monitoring EOG, OECT‐based complementary inverters, organic electrochemical transistors

## Abstract

Organic electrochemical transistors (OECTs) and their related circuits have emerged as a promising platform for biosensors and neuromorphic electronics, benefiting from their sub‐1 V operation voltage, flexibility, biocompatibility, etc. However, operation instability, induced by complex microstructure variations and undesired side reactions during repeated redox processes, poses tremendous challenges for reliable and robust functions. Here, a dynamic modulating system is presented that can actively control the working condition of an OECT‐based inverter and maintain high‐voltage amplification capability through real‐time voltage transfer characteristic scanning and operating voltage adjustment. Especially, under system modulation, the inverter maintains a high‐voltage amplification capability with a voltage gain >34.58 V V^−1^. While without modulation (i.e., at a fixed input voltage), the voltage gain rapidly deteriorates to 3.11 V V^−1^. Thereafter, stretchable complementary circuits are fabricated and integrated with this system to enable high‐fidelity in situ monitoring of the electrooculogram with >32.59 dB signal‐to‐noise ratio for more than 90 min, thus establishing a reliable wearable biosensing method. This work provides a new strategy to enable highly stable operation of bioelectronics with devices holding inferior stabilities.

## Graphical abstract

This work presents a dynamic gain modulation system for inverter‐based in situ electrophysiological monitoring, enabling long‐term stable signal amplification via real‐time driving voltage adjustment. Moreover, stretchable complementary circuits (inverters, NAND, and NOR gates) are achieved based on a flexible PET substrate and a hexagonal network structure architecture, which further enables high‐fidelity in situ EOG monitoring for 90 min.

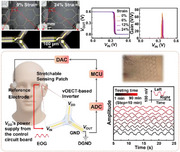

## 1 Introduction

Organic electrochemical transistors (OECTs) demonstrate widespread advantages in bioelectronics,^[^ ^1^, ^2^, ^3^, ^4^ ^]^ biosensors,^[^ ^5^, ^6^, ^7^, ^8^, ^9^ ^]^ and neuromorphic electronics,^[^ ^10^, ^11^, ^12^, ^13^, ^14^ ^]^ due to their sub‐1 V operation, high transconductance (*g*_m_), and flexibility. Therefore, OECTs and their related circuits have demonstrated high‐precision monitoring of diverse physiological signals, including electroencephalography (EEG), electrocardiography (ECG), electrooculography (EOG), electromyography, etc,^[^ ^15^, ^16^, ^17^, ^18^, ^19^ ^]^ accelerating wearable medical diagnostics development. Despite their promises, OECT‐based bio‐signal acquisition faces two critical limitations. First, the output current range (pA to mA) of OECTs surpasses conventional analog‐to‐digital converters (ADC) limits, and requires transimpedance amplifiers‐based conversion, which then introduces noise penalty through discrete components (resistors/capacitors/op‐amps), degrading acquisition accuracy.^[^ ^19^, ^20^, ^21^ ^]^ Second, operational instability and poor storage reliability hinder electronic compatibility, restricting widespread adoption and commercialization.^[^ ^22^, ^23^ ^]^

Consequently, inverters, especially complementary inverters, based on OECTs, present an ideal solution to the output current range challenge. By leveraging the high‐voltage gain of its linear region (transition zone), it enables efficient amplification of weak input signals at low supply voltages (*V*_DD_ < 1 V).^[^ ^24^, ^25^, ^26^, ^27^ ^]^ Compared to single OECT, complementary inverters exhibit superior performances, where the direct voltage signal output is compatible with ADC sampling, significantly reducing system thermal noise interference.^[^ ^15^, ^27^ ^]^ Moreover, the high‐voltage gain makes them ideal to amplify small physiological signals when biasing the inverter in the transition range.^[^ ^26^, ^28^, ^29^, ^30^ ^]^ These characteristics make OECT‐based inverters an ideal platform for physiological signal detection.^[^ ^31^, ^32^, ^33^, ^34^, ^35^ ^]^

Nevertheless, the complementary inverters for bioelectrical signal sensing still face the stability bottleneck. Due to the electrochemical side reactions and current stress effects during ion doping/dedoping processes of the OECT channel, the performance of OECTs would degrade much faster than conventional transistors.^[^ ^36^, ^37^, ^38^, ^39^, ^40^, ^41^ ^]^ Specifically, these phenomena induce irreversible degradation of the microstructure and chemical composition of the organic mixed ionic‐electronic (semi)conductor (OMIEC) channel,^[^ ^40^, ^42^ ^]^ leading to continuous drift in OECT transfer characteristics. Consequently, complex shifts in the voltage transfer characteristics (VTCs) are observed, along with fluctuations in the gain coefficient. Therefore, under a fixed bias input voltage (*V*_IN_), it would be impossible to enable the transistor to work stably in the transition range. Thus, the amplification efficiency for physiological signals is significantly reduced.

**Figure 1:**
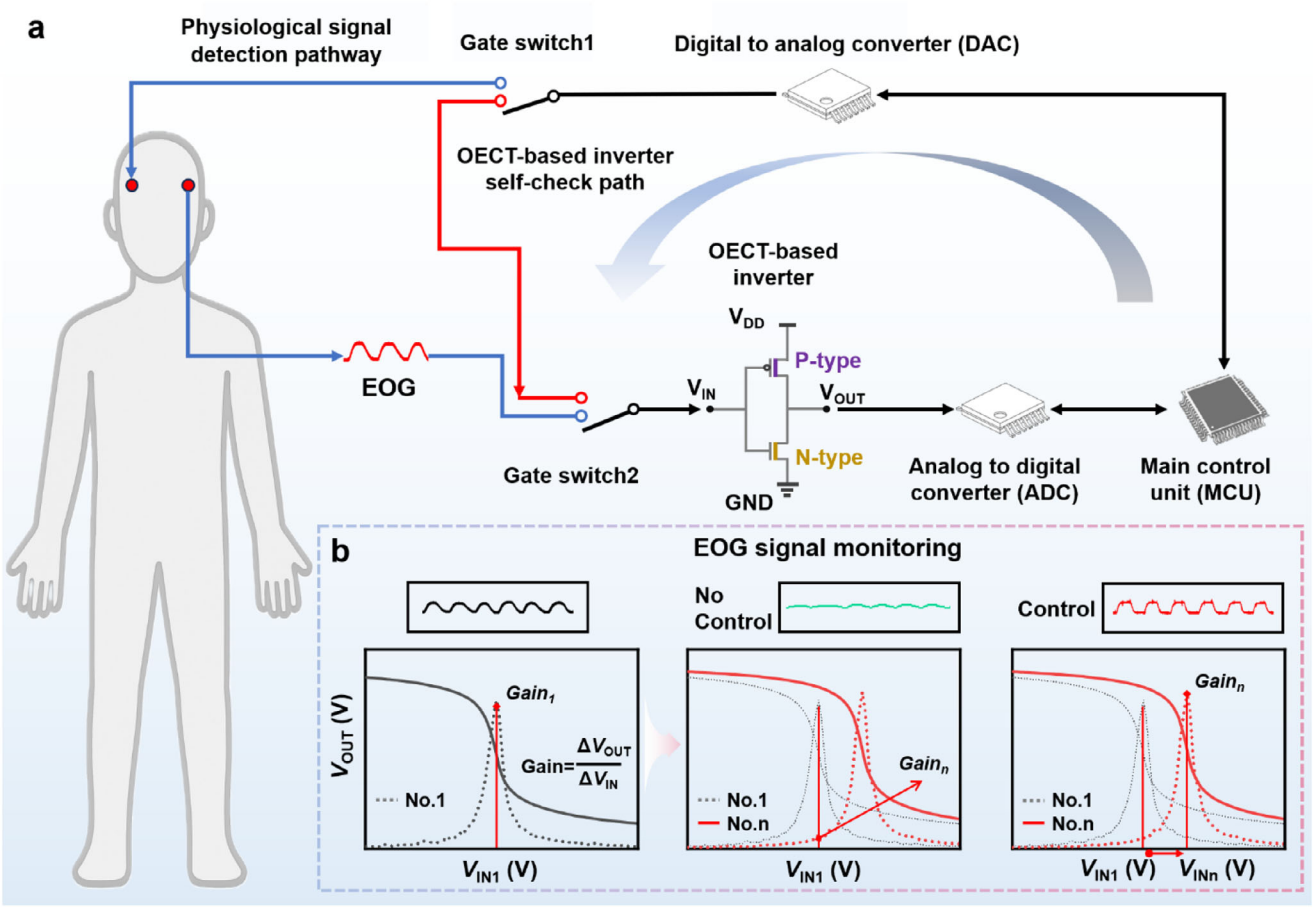
Schematic diagram of the dynamic gain modulating system for the OECT‐based inverter. a) The inverter undergoes self‐checking via the modulation system to determine *V*_m_. First, gate switches 1 and 2 are turned to the self‐check path to determine *V*_m_. Then, a DAC precisely adjusts *V*_IN_ to *V*_m_ and switches to the physiological signal detection pathway for high‐voltage gain sensing. b) Demonstration of the obtained EOG signals with and without the dynamic gain modulation.

Here, a dynamic modulating system is developed to ensure that the inverter is always driving around the voltage that yields the maximum voltage gain (*V*_m_). In this process, the system first locates *V*_m_ by scanning VTC through a self‐check path that consists of an OECT‐based inverter, subsequently controls *V*_IN_ to match the identified *V*_m_, and amplifies the physiological signal at this optimized operating condition through the physiological signal detection pathway (**Figure** **1**). This detection‐control‐amplification closed‐loop feedback system operates continuously throughout the entire measurement process. Especially, the introduction of the dynamic modulating system maintains high‐voltage amplification capability by keeping voltage gain >34.58 V/V. While without control (inverters work under a fixed *V*_IN_), the voltage gain rapidly decreases to 3.11 V/V within 10 min. Furthermore, stretchable complementary circuits are fabricated and integrated with this system, achieving high‐fidelity in situ monitoring of human EOG signals. This work not only mitigates signal attenuation from OECT electrochemical instability but also further establishes a new paradigm for precision sensing in wearable electronics.

## 2 Results and Discussions

### 2.1 Performance of OECT‐Based Complementary Inverters

**Figure 2:**
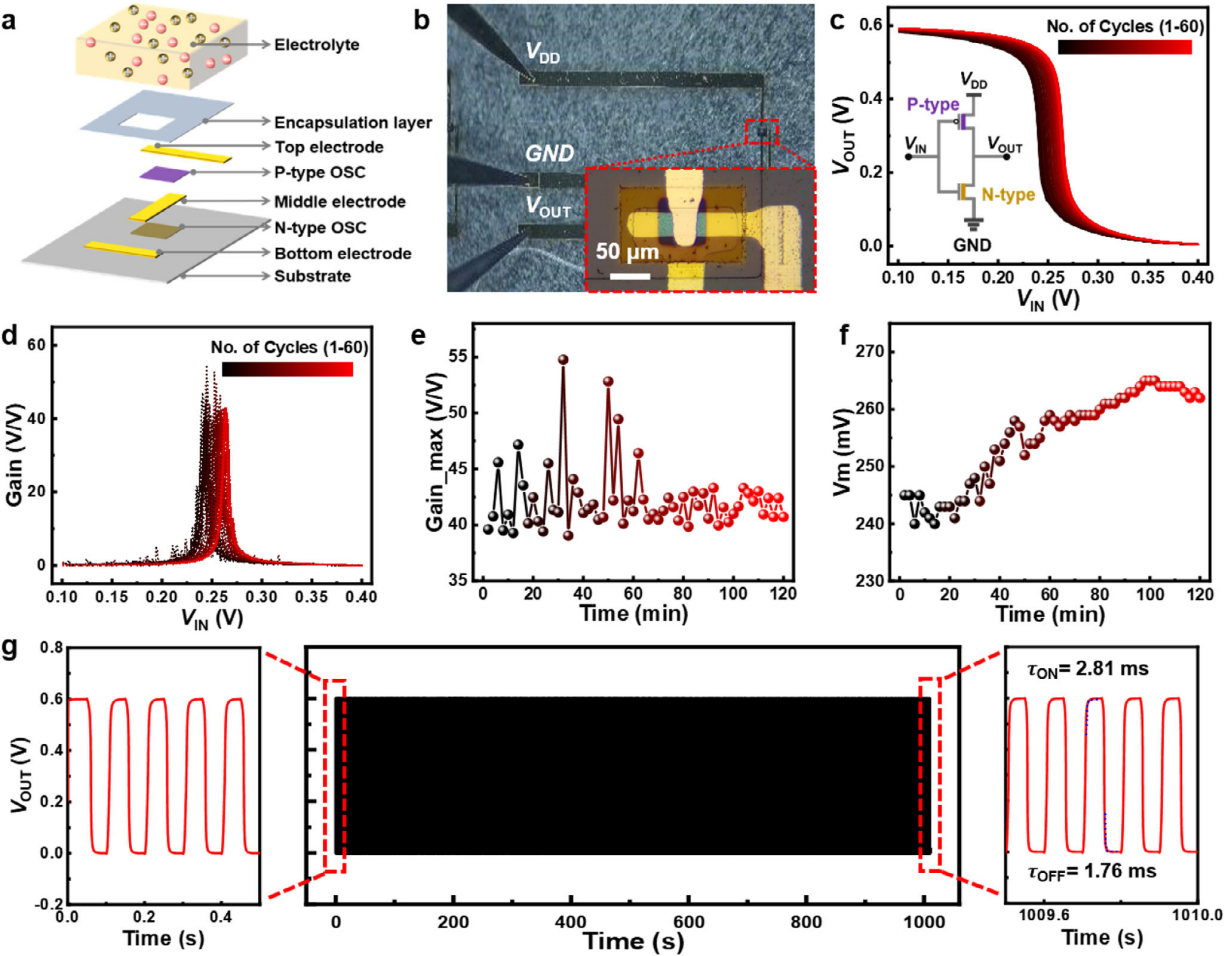
Structure and performance of the OECT‐based complementary inverter. a) Exploded schematic view, and b) optical micrograph of the OECT‐based inverter; c) VTC curves and d) corresponding gain curves for repeated tests up to 60 cycles (*V*_DD_=0.6 V); e) Extracted voltage gain and f) corresponding *V*_m_; g) Cycling stability of the inverter (*V*_DD_=0.6 V) with enlarged first 1–5 and last 10 095–10 100 cycles. The channel dimensions of the n‐ and p‐type OECTs are 120 × 30 and 30 × 30 µm^2^, respectively.

The complementary inverter comprises two vertically stacked OECTs, where a p‐type poly(2,5‐dipentaethyleneglycol‐3,6‐di(thiophen‐2‐yl)‐2,5‐dihydropyrrolo[3,4‐c]pyrrole‐1,4‐dione‐alt‐2,5‐bis(3‐triethyleneglycoloxythiophen‐2‐yl) (gDPP‐g2T)‐based OECT is located on top of an n‐type poly(benzimidazobenzophenanthroline) (BBL)‐based OECT (**Figure** **2**a). The detailed fabrication process can be found in Figure S1 (Supporting Information) and the Experimental Section. The circuit configuration incorporates: a bottom‐grounded electrode as ground (GND), an intermediate output electrode as output voltage (*V*_OUT_), a top electrode as *V*_DD_, along with a silver/silver chloride (Ag/AgCl) electrode immersed in the electrolyte as *V*_IN_. Electrical characterization was conducted via probe station interfacing with peripheral circuitry (Figure 2b). As shown in Figure S2 (Supporting Information), p/n‐type OECTs show on‐current (*I*_ON_) of 2.59 ± 0.31 and 2.42 ± 0.24 mA, respectively, along with balanced *g*_m_ of 13.76 ± 3.71 and 7.41 ± 1.58 mS, respectively. Moreover, both types of OECTs show switching speed in the range of 1–13 ms, along with turn‐on voltages (*V*_ON_) of 0.08 and ‐0.17 V obtained in p‐type and n‐type OECTs, respectively. Specifically, a larger n‐type channel size was designed to enhance *I*_ON_ and *g*_m_, thereby balancing performance with the p‐type OECT. Such balanced transfer characteristics indicate that inverters based on such p‐/n‐type OECT pairs would lead to balanced inverter properties. Next, the performance of the inverter is characterized, which exhibits distinct switching capability under *V*_DD_ of 0.2–0.6 V (Figure S3, Supporting Information). Voltage gain >50 V/V and power dissipation <1 mW are achieved under *V*_DD_ of 0.6 V. Additionally, *V*_m_ of the inverter lies around *V*_DD_/2, enabling effective VTC even under low *V*_DD_ of 0.2 V. Furthermore, the inverter reveals a transient response time <3 ms (*τ*_ON_═1.76 ± 0.18 ms, *τ*_OFF_═2.20 ± 0.28 ms) and can be operated under a frequency of 50 Hz.

On the other hand, VTC and voltage gain curves of such an inverter show a sustained shift after repeated cycling, as shown in Figure 2c,d. During this process, the voltage gain fluctuates between 39.05 and 54.47 V/V with an average value of 42.23 V/V (Figure 2e), while *V*_m_ continuously increases from 240 to 265 mV (Figure 2f). This instability or shift in performance may stem from irreversible microstructural and/or chemical degradation of the OMIEC channel due to repeated ion doping/dedoping processes, or electrolyte evaporation and interface degeneration during operation. Nevertheless, the fabricated inverter maintains high gain (>39 V V^−1^, Figure 2e) over an extended period and demonstrates more than 10 000 switching cycles (cycling frequency of 10 Hz) with no degradation on *V*_OUT_ level and transient times (Figure 2g), underscoring its practical viability for real‐world applications.

### 2.2 Dynamic Modulating System for OECT‐Based Complementary Inverters

**Figure 3:**
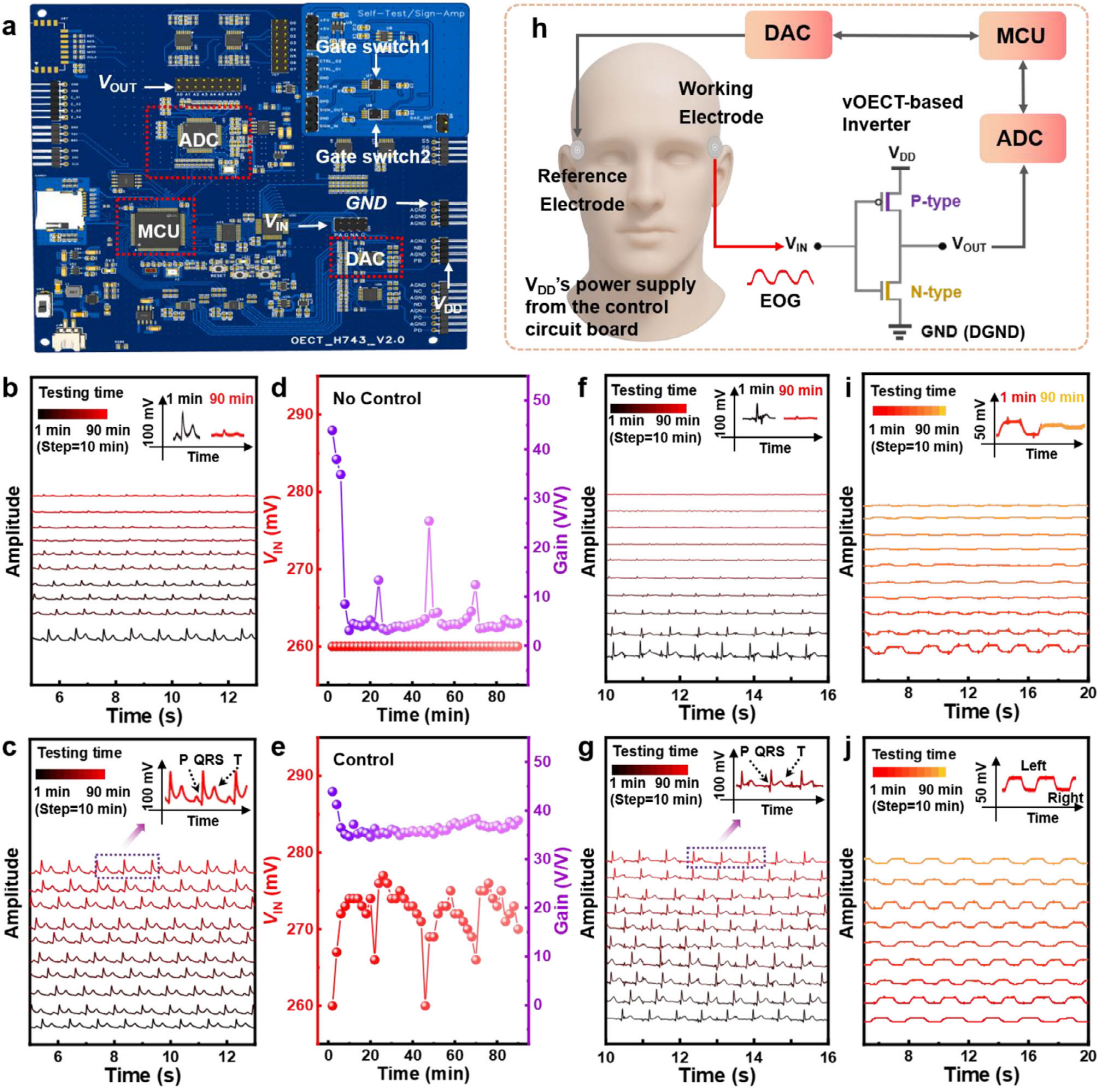
Dynamic modulating system setup and biological signal detection with OECT‐based complementary inverters. a) Schematic illustration of the dynamic gain modulation system. Comparison of simulated ECG signals monitored b) without and c) with the dynamic modulating system. Corresponding inverter *V*_m_ and voltage gain values under d) a fixed *V*_IN_ of 260 mV and e) active modulating *V*_IN_. Real‐time human ECG signals recorded without f) and with g) the modulating system. h) Working diagram of the OECT‐based inverter for EOG signal detection. Real‐time human EOG signals recorded without i) and with j) the modulating system. A 0.05–30 Hz bandpass filter and baseline leveling are applied for EOG and ECG signals.

The dynamic modulating system performs continuous output monitoring and feedback‐controlled *V*_IN_ adjustment to maintain operation at the optimal *V*_m_ (*V*_m1_, *V*_m2_, … *V*_mn_). It primarily relies on the sequential operation of three core components (**Figure**  **3**a; Figure S4, Supporting Information). First, the signal acquisition module serves as the front end, where a high‐resolution 24‐bit ADC (AD7768‐1) continuously captures the *V*_OUT_ of the complementary inverters and converts it into a digital signal in real time. The digital signal is then transmitted to the processing unit, which is centered around an STM32H743VIT6 microcontroller. This unit determines *V*_m_ through signal analysis and coordinates the entire optimization process. Last, the voltage control module, constructed using a 16‐bit DAC (DAC8562), precisely adjusts the inverter bias *V*_IN_ within the 0–600 mV range with a 1 mV step, to monitor the physiological electrical signals. After a period of signal detection (time interval can be adjusted based on need), the system turns back to the self‐check path to determine *V*_m_ (≈15 s), and then reverts to the signal detection path. The 15‐s self‐check procedure would not affect subsequent signal monitoring (Figure  S4, Supporting Information). Detailed system construction and process flow can be found in Figure S5 (Supporting Information).

To evaluate system performance, simulated ECG signals are first monitored through the inverter under controlled or uncontrolled conditions. As shown in Figure 3b, without the modulating system, progressive signal attenuation and severe waveform distortion are observed as the monitoring continues, where the signal‐to‐noise ratio (SNR) decreases from 28.98 ± 2.43 dB (1st min) to 12.32 ± 1.13 dB (after 90 min) (Table S1, Supporting Information). In contrast, with the modulating system, high‐quality ECG signals, exhibiting clear P‐waves, QRS complexes, and T‐waves, are monitored with high fidelity (SNR ≥ 28.73 ± 2.16 dB) for continuous monitoring of 90 min (Figure 3c; Table S1, Supporting Information). VTCs during the continuous monitoring of the simulated ECG signals in both cases are demonstrated (Figure S6, Supporting Information). Here, varying *V*_m_ with or without the modulating system under continuous testing bias is identified. It is worth noting that, without control, the inverter would be biased under a constant *V*_IN_. As shown in Figure 3d, a *V*_IN_ of 260 mV is applied. Initially, the inverter enables a high‐voltage gain of 43.92 V/V. However, due to the shifted *V*_m_, the obtained voltage gain under driving *V*_IN_ rapidly decreases to < 10 V/V within 10 min, severely affecting the amplification of targeting signals. In contrast, with the aid of the dynamic modulating system, *V*_IN_ varies actively to maintain a high‐voltage gain (>34.58 V/V) via real‐time *V*_m_ tracking (Figure 3e). To capture real‐time human ECG signals, the device structure is further optimized—including the dimensions of the patterned semiconductor and the encapsulation exposed in the channel region—to reduce parasitic capacitance and enhance the response frequency (Figure S7a, Supporting Information). Voltage gains up to 135 V/V and operating frequencies up to 333 Hz are achieved (Figure S7b–f, Supporting Information). Subsequently, without the modulation system, the captured ECG signal gradually attenuates along with severe waveform distortion as the motoring continues, showing decreasing SNR from 27.15 ± 2.61 (1st min) to 8.79 ± 1.23 dB (after 90 min) (Figure 3f; Table S2, Supporting Information). Conversely, with the modulation system, high‐quality ECG signals (SNR≥27.32 ± 2.93 dB), exhibiting clear P‐waves, QRS complexes, and T‐waves, are recorded for up to 90 min (Figure 3g; Figure S8, Supporting Information). Additionally, an amplified voltage variation of up to 41 mV is successfully acquired through real‐time human ECG monitoring using the modulation system.

Next, by attaching two electrode patches (3 M 2244) on the left and right sides of the human eye, the potential difference between the cornea and retina caused by the left‐right movement of the eyeball (horizontal EOG) is measured (Figure 3h–j). It is shown that without the modulation system, the signal gradually attenuates and severe waveform distortion is observed as the motoring continues, with the SNR decreasing from 27.53 ± 2.89 dB (1st min) to 10.58 ± 1.39 dB (after 90 min) (Figure 3i; Table S3, Supporting Information). However, with the modulating system, high‐quality EOG signals are captured with high signal fidelity (SNR ≥28.50 ± 3.08 dB) for continuous monitoring of 90 min (Figure 3j). Moreover, an amplified voltage variation of up to 23 mV is achieved by the system in monitoring horizontal eye movement. By dynamically modulating *V*_m_, this system significantly enhances the reliability and accuracy of long‐term bioelectrical signal (ECG/EOG) monitoring.

### 2.3 OECT‐Based Stretchable Circuits and In Situ EOG Monitoring

**Figure 4:**
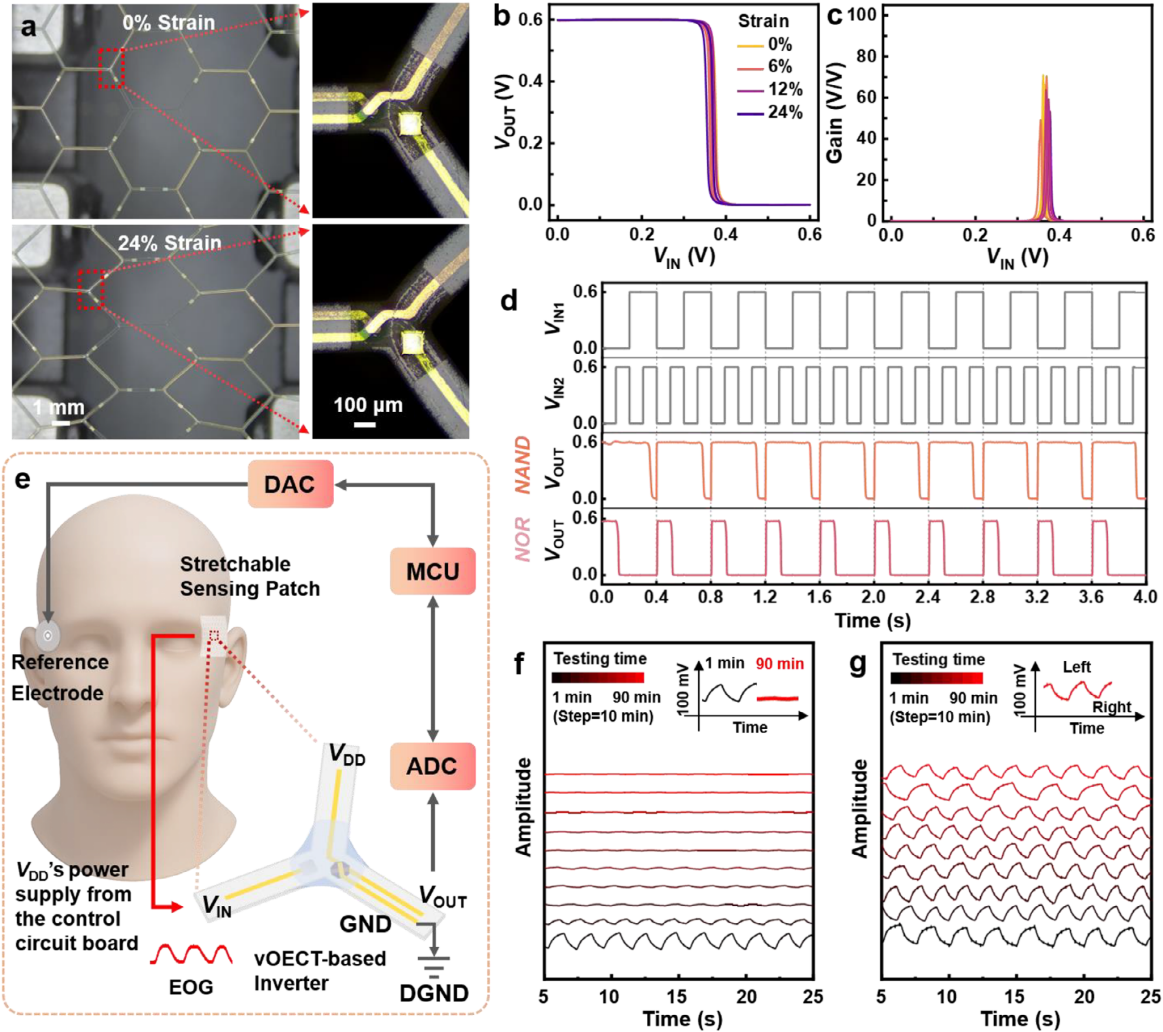
OECT‐based stretchable complementary circuits and in situ EOG monitoring. a) Optical microscopy image of the device under 0 and 24% tensile strain, demonstrating its mechanical robustness. b) Inverter VTC and c) corresponding gain under different strains (0%, 6%, 12%, and 24%). d) Logic functions (NAND and NOR gates) with *V*_DD_ of 0.6 V. e) Schematic illustration of the in situ EOG monitoring using the stretchable inverter patch. 90 mins of EOG signal monitoring f) without and g) with the modulating system. A 0.05–30 Hz bandpass filter and baseline leveling are applied for EOG signals.

Next, stretchable complementary circuits based on OECTs are fabricated, along with the demonstration of their potential for in situ EOG monitoring. To achieve stretchability, flexible polyethylene terephthalate (PET) substrates are applied with a hexagonal honeycomb grid structure. This design enables macroscopic stretchability through the flexure and rotation of the mesh units, despite the non‐ductile nature of PET itself.^[^ ^43^, ^44^ ^]^ The overall circuit diagram is redesigned to ensure all functional layers lie on the grid structure, which allows for effective stretching of the grid while not inducing obvious strains on functional layers (**Figure**  **4**a). Detailed preparation processes can be found in Figure S9 (Supporting Information) and the Experimental Section.

First, a comparative analysis of inverter configurations revealed that with different electrolytes and gate configurations, excellent VTC with *V*_m_ maintained at ≈1/2*V*_DD_ can still be obtained with the grid structure (Figure S10, Supporting Information). Furthermore, under 0–24% strain (Figure S11, Supporting Information), p/n‐type OECT demonstrated that *I*_ON_ only decreased from 0.42 ± 0.03/0.28 ± 0.02 to 0.08 ± 0.01/0.21 ± 0.02 mA, along with a decrease in *g*_m_ from 3.08 ± 0.24/2.16 ± 0.15 to 1.00 ± 0.12/1.44 ± 0.08 mS (Figure S12a,b, Supporting Information). Despite those, the OECT‐based inverter still achieves high‐voltage gain (>50 V/V) and stable *V*_m_ under strain up to 24% (Figure 4b,c). Notably, even at 24% strain, the inverter maintains low power dissipation <0.1 mW (Figure S12c, Supporting Information), fast transient response < 6 ms (*τ*_ON_═2.89 ± 0.14 ms, *τ*_OFF_═5.86 ± 0.39 ms), and stable operation under a frequency of more than 20 Hz (Figure S12d, Supporting Information). More than 10 000 stable switching cycles (cycling frequency of 10 Hz) with no degradation on the *V*_OUT_ level (Figure  S12e, Supporting Information) also reveal good long‐term working capability of such stretchable inverters. The device also exhibits excellent stability during 1000 dynamic tensile cycles (with a strain of 0–24% over 1000 s) (Figure S13, Supporting Information), and maintains good performance across 20–50  °C, with the gain increasing from 73 to 89 V/V, demonstrating reliable operation within this temperature range (Figure S14, Supporting Information). Thereafter, complementary NAND/NOR logic gates are further constructed (Figures S15and S16, Supporting Information), which exhibit characteristic truth‐table behavior under 24% strain. Under 0.6 V *V*_DD_ and 0–0.6 V square‐wave *V*_IN_ of 2.5 Hz (*V*_IN1_) and 5 Hz (*V*_IN2_), the NAND gate maintained a logic “1” output unless both inputs simultaneously reach “1” state, while the NOR gate output switched to “1” only when both inputs are at “0” state (Figure 4d).

For in situ EOG monitoring, typical periorbital strains during eye and eyelid movement remain below 15%.^[^ ^45^ ^]^ A stretchability margin of 24% is selected to exceed this strain range with a sufficient safety factor, thereby ensuring signal integrity and robustness against motion artifacts. The stretchable inverter patch can be easily positioned on the right side of the right eye (with reference electrode located on the left side of the left eye) to capture high‐fidelity EOG signals with a dynamic gain modulating system (Figure 4e; Figure S17, Supporting Information). 90‐min in situ EOG monitoring is then conducted (Figure 4f,g). Without the modulating system, the amplitude rapidly decayed after the first interval, despite an initial peak of ≈47 mV, where the SNR decreases from 32.93 ± 4.51 dB (1st min) to 12.21 ± 1.28 dB (after 90 min) (Figure 4f; Table S4, Supporting Information). In contrast, the horizontal EOG amplitude with the modulating system remained stable at ≈48 mV throughout the recording process (SNR >32.53 ± 3.86 dB) (Figure 4g; Table S4, Supporting Information).

## 3 Conclusion

In summary, this work reports an inverter‐based dynamic gain modulating system for in situ electrophysiological monitoring. The modulation system demonstrated here is achieved by tracking *V*_m_ of the inverter in real time to serve as the operating voltage for physiological signal monitoring, which effectively enables long‐term stable amplification of targeting signals. Meanwhile, stretchable complementary circuits (inverters, NAND, and NOR gates) are achieved based on a flexible substrate (PET) and a hexagonal network structure architecture, and provide for high‐fidelity in situ EOG monitoring for 90 min. These results indicate that combining the stretchable circuits with a smart modulating system can provide an effective strategy for long‐term biopotential monitoring applications. This work also offers new possibilities for next‐generation healthcare monitoring and assistive technologies.

## 4 Experimental Section

### OECT‐Based Inverter Fabrication

The inverter fabrication process (Figure S1, Supporting Information) begins with the thermal evaporation of Cr/Au bottom electrodes (3 nm Cr/50 nm Au, deposition rate: 0.1/0.2–1.5 Å s^−1^) through a shadow mask on pre‐cleaned glass substrates, serving as the GND. Subsequently, an n‐type channel is formed by spin‐coating (3000 rpm, 1 min) methanesulfonic acid‐dissolved BBL solution (15 mg mL^−1^, Sigma–Aldrich, stirred overnight), followed by a 15‐min DI water immersion to remove residual solvent and N_2_ drying. The channel was then patterned using laser etching (ProtoLaser R4, LPKF Inc.) with the following optimized parameters: frequency of 500 kHz, power of 0.12 W, scan speed of 1500 mm s^−1^, pitch of 5 µm, and 1 cycle. An intermediate Au electrode (50 nm, 0.2–1.0 Å s^−1^) was evaporated for *V*_OUT_. For the p‐type layer, a gDPP‐g2T solution (20 mg mL^−1^ in chloroform with crosslinker, 10:1 in a mass ratio, synthesized via previously reported methods^[^ ^46^, ^47^, ^48^ ^]^) was spin‐coated (3000 rpm, 20 s), UV‐crosslinked (365 nm, 450 mW cm^−^^2^, 360 s), and cleaned with chloroform. The top Au electrode (*V*_DD_) was deposited using identical parameters, with the substrate maintained at 5 °C during the above evaporation. Finally, SU‐8 (2002) encapsulation was achieved by spin‐coating (2000 rpm, 1 min), soft baking (95 °C, 1 min), UV exposure (30 s), development (30 s), and IPA rinsing. Device characterization was performed using 0.01 m PBS electrolyte with an Ag/AgCl floating gate electrode.

### OECT‐Based Stretchable Circuits Fabrication

Inverter: As illustrated in Figure S9 (Supporting Information), the fabrication involves sequential thermal evaporation of Cr/Au electrodes on PET substrates (0.03 mm). The p‐type (gDPP‐g2T) and n‐type (Homo‐Poly(2,5‐dipentaethyleneglycol‐3,6‐di(thiophen‐2‐yl)‐2,5‐dihydropyrrolo[3,4‐c]pyrrole‐1,4‐dione) (Homo‐gDPP), synthesized via previously reported methods^[^ ^46^, ^47^, ^48^ ^]^) channels were patterned through photolithography after spin‐coating their respective solutions (20 mg mL^−1^ with crosslinker, 10:1 in a mass ratio). Silver patches (80 × 80 µm^2^) were printed on gates using the XTPL Delta Printing System (5 µm nozzle) and laser‐annealed (0.2 W, 100 kHz, 200 mm s^−1^, 6 cycles). SU‐8 (2002) encapsulation follows the aforementioned protocol. The PET substrate was laser‐patterned (2.5 W, 100 kHz, 200 mm s^−1^, 60 cycles) into honeycomb structures. The devices were completed with polyethylene glycol (PEG)‐Lithium chloride (LiCl) electrolyte deposition and vacuum drying (45 °C, 1 h). NAND and NOR Gates: The logic gates (Figure  S16, Supporting Information) were fabricated on PET substrates through a similar process flow. After electrode evaporation, the p‐type (gDPP‐g2T) and n‐type (Homo‐gDPP) channels are sequentially patterned. Silver gate contacts and laser annealing was performed as described above. The honeycomb‐structured PET substrates are prepared using identical laser parameters. Two separate *V*_IN_ regions are created by depositing PEG‐LiCl electrolyte as *V*_IN1_ and *V*_IN2_ inputs, followed by vacuum drying (45 °C, 1 h).

### Device Characterization

The transfer characteristics and transient response of the transistors were evaluated using 0.01 m PBS electrolyte with an Ag/AgCl floating gate electrode (Figure S18, Supporting Information). The OECTs based on BBL (gDPP‐g2T and Homo‐gDPP) were characterized using a probe station (Chengdu Chip Testing Technology Co., Ltd.) connected to a semiconductor parameter analyzer (FS‐Pro, PDA). The functionality of NAND and NOR logic gates was tested using a probe station connected to a semiconductor parameter analyzer (FS‐Pro, PDA) and a DC voltage source. A constant *V*_DD_ of 0.6 V was applied by the DC voltage source, while square‐wave input pulses (0.0 to 0.6 V) for *V*_IN1_ (2.5 Hz) and *V*_IN2_ (5 Hz) were supplied by the semiconductor parameter analyzer, with *V*_OUT_ monitored in real time. To maintain PBS electrolyte stability, the testing area was covered with a PDMS mold to minimize solvent evaporation. All experiments were conducted under ambient conditions.

### Human EOG/ECG Monitoring

A 22‐year‐old healthy male participated in the experiment, and all testing procedures were performed safely without any external stimuli. Before the experiment, human subjects were trained on the experimental protocol, including horizontal eye movements, and were told not to talk and not to have any head movements to avoid interference from other body movements and the EOG signal. For ex situ monitoring, the disposable electrode patches (3 M 2244) were placed on the left and right sides of the human eye, respectively. The inverter is then connected to the peripheral circuit system via the probe station. The left electrode patch was connected to the ADC terminal, and the right electrode patch was directly connected to the *V*_IN_ of the mentioned inverter. *V*_DD_ was powered by the dynamic gain modulation system. The inverter's GND terminal is connected to the control circuit board's GND, sharing a common GND with other components. Both the inverter and DAC are connected to the analog GND (AGND), while the main control unit interfaces with the digital GND (DGND). The ADC's analog front‐end uses AGND, whereas its digital communication pins connect to DGND. AGND and DGND are coupled via a series resistor to minimize digital‐to‐analog signal interference. Subsequently, in a relaxed state, the subject was asked to gaze horizontally and then was asked to move their eyes to the right and then to the left. This procedure was repeated during the measurement. The amplified EOG signal was recorded by monitoring *V*_OUT_ using a dynamic gain modulating system. For real‐time vitro ECG monitoring, two electrode patches were positioned 2 cm below the nipples, secured with medical tape to maintain stable contact and minimize motion artifacts. Volunteers with low body fat were selected and instructed to remain seated comfortably while breathing steadily. The skin was pre‐treated using soap and alcohol cleansing, followed by mild exfoliation. The same testing protocol as the ex situ measurement was then conducted. For in situ EOG monitoring, the inverter was attached to the left side of the human eye by adopting PBS electrolyte (Figure S17, Supporting Information). In addition, the external copper wires connecting the system were first fixed to the electrode leads at *V*_DD_, *V*_IN_, *V*_OUT_, and GND of the inverter, respectively, through solder paste, and then dripping silver paste followed by vacuum drying (80 °C, 2 h). Subsequently, the same testing procedure was conducted as that of the ex‐situ one.

### SNR Calculation

To calculate the SNR of sampled signals with and without a dynamic gain optimization system, the effective power in the frequency range of 0.05 to 30 Hz is calculated after applying a 0.05 Hz high‐pass filter to process baseline drift, as well as the noise power for frequencies greater than 30 Hz.

(1) $$power_{signal}=\sum {\left|fft\_result\left[fft_{freq}>0.05andfft_{freq}\leq 30\right]\right|}^{2}$$

(2) $$power_{noise}=\sum {\left|fft\_result\left[fft_{freq}>30\right]\right|}^{2}$$

Subsequently, the SNR can be calculated using the obtained signal power and noise power.^[^ ^49^ ^]^

(3) $$SNR=10\log_{10}\frac{power_{signal}}{power_{noise}}$$

### Statistical Analysis

Data with conspicuous outliers were excluded by the interquartile range (IQR) and 1.5 times the IQR. Data in the manuscript are presented as mean ± standard deviation (SD). The sample size (n) for each statistical analysis comprises no less than five valid data points, representing the number of independent replicates. Statistical analyses were performed using Excel.

## Conflict of Interest

The authors declare no conflict of interest.

## Supporting information



Supporting Information

## Data Availability

The data that support the findings of this study are available from the corresponding author upon reasonable request.
