# Supplementary material for: Dynamic Modulation of OECT‐Based Inverters for In Situ Electrophysiological Monitoring
Source: Adv Sci (Weinh). 2025 Oct 17;13(5):e12755. doi: 10.1002/advs.202512755 (PMC12850065; doi:10.1002/advs.202512755)
Supplement: Supplementary file 1 — Supporting Information [file ADVS-13-e12755-s001.docx]

Supporting Information

Dynamic Modulation of OECT-Based Inverters for In-Situ Electrophysiological Monitoring

Guohong Hu, Qijun Cai, Zhenglei Liu, Rongsheng Zeng, Liang-Wen Feng, Jianhua Chen, Shiji Xiahou*, Wei Huang*


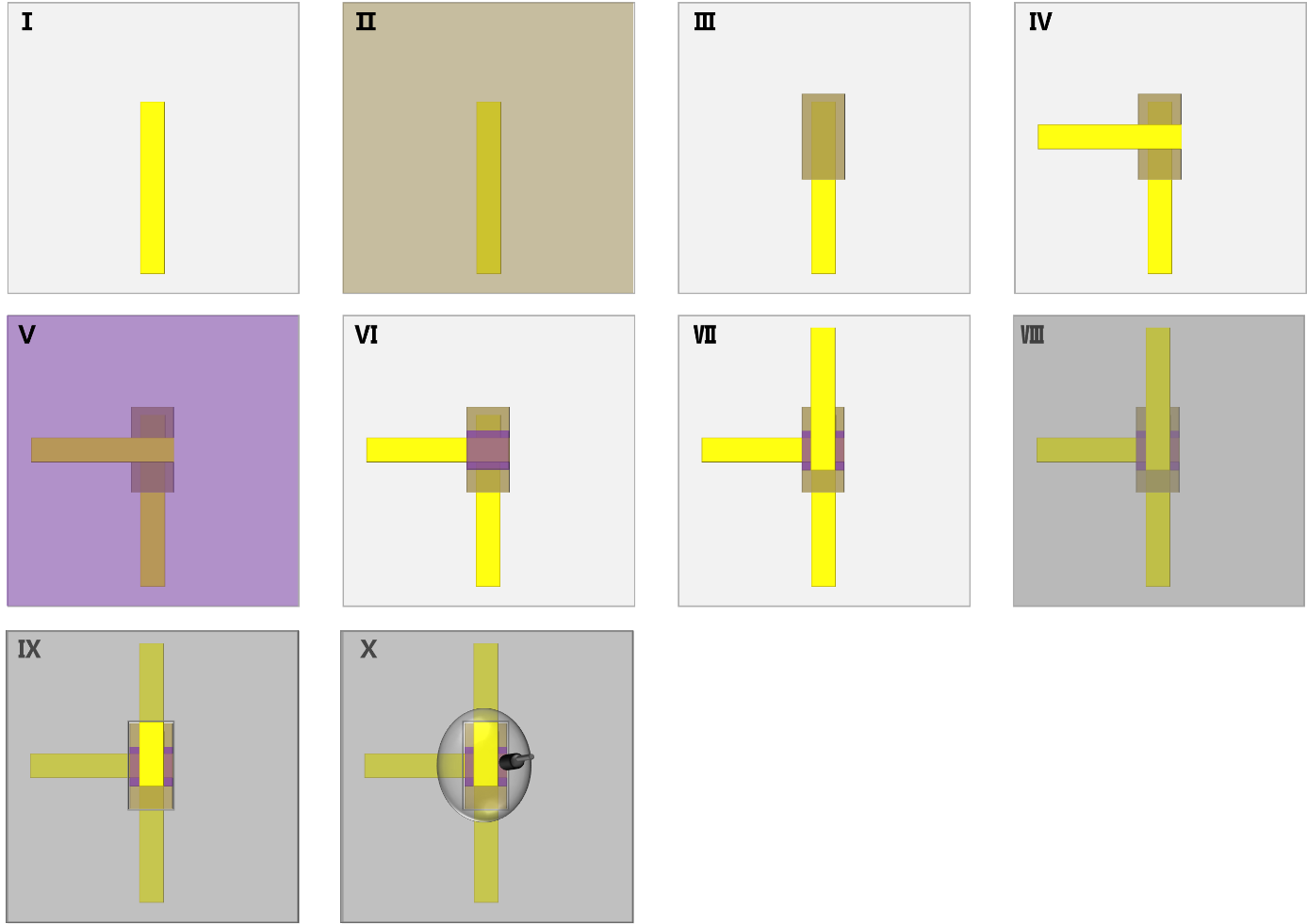


**Figure S1.** Fabrication process of the OECT-based complementary inverter. Ⅰ) Bottom electrode deposition, Ⅱ) N-type semiconductor (BBL) deposition, Ⅲ) N-type channel patterning, Ⅳ) Middle electrode deposition, Ⅴ) P-type semiconductor (gDPP-g2T) deposition, Ⅵ) P-type channel patterning, Ⅶ) Top electrode deposition, Ⅷ) Photoresist (SU8-2002) deposition, Ⅸ) Photoresist development, Ⅹ)Electrolyte and Ag/AgCl floating gate application.


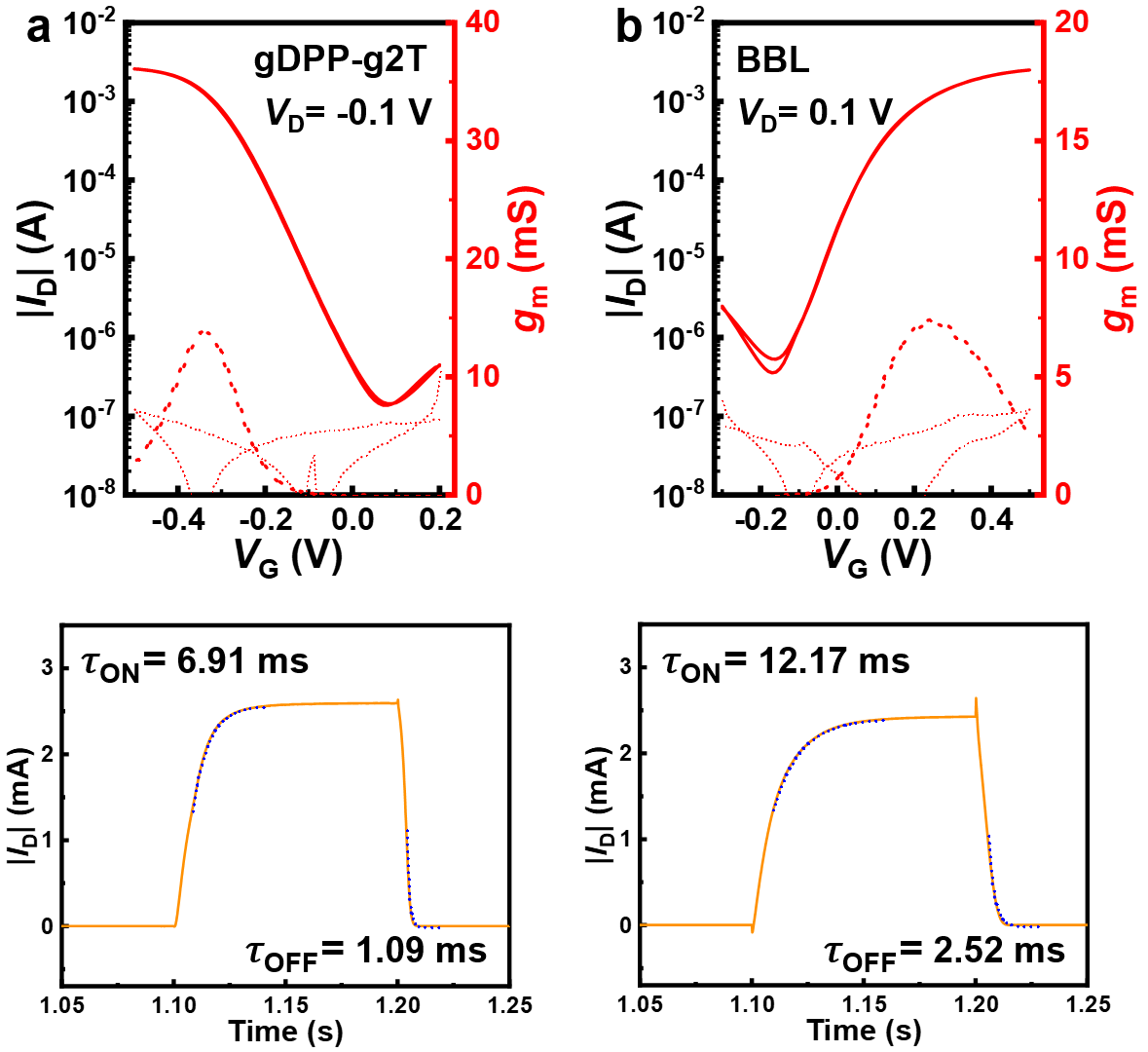


**Figure S2.** a) Transfer characteristics (top) and transient response (bottom) of a p-type OECT in the inverter; b) Transfer characteristics (top) and transient response (bottom) of an n-type OECT in the inverter. The channel dimensions of the p-type and n-type OECTs are 30 × 30 μm² and 120 × 30 μm², respectively.


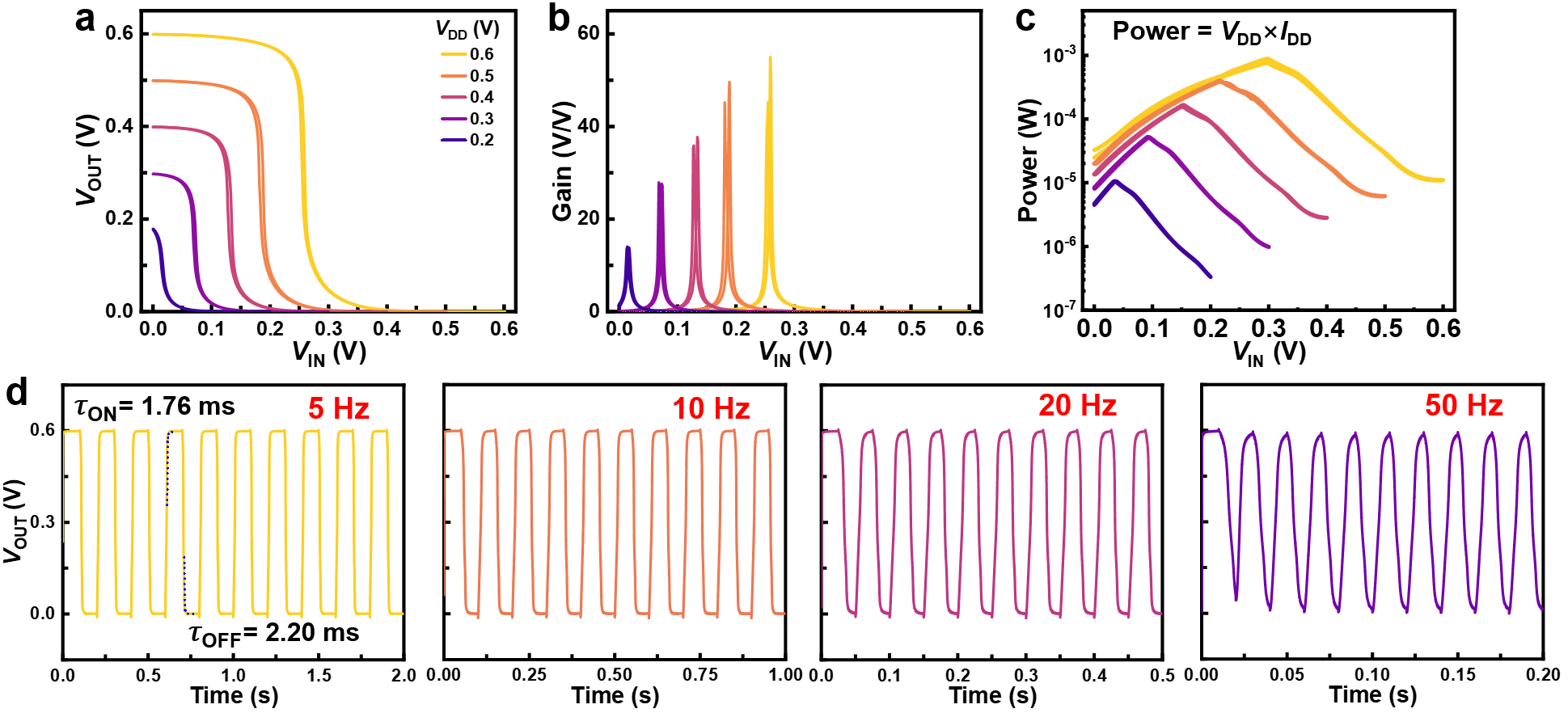


**Figure S3.** a) Voltage transfer characteristic, b) corresponding voltage gain, and c) power consumption of the inverter at *V*_DD_ = 0.2, 0.3, 0.4, 0.5, and 0.6 V; d) Transient response of the inverter under different driving frequencies.


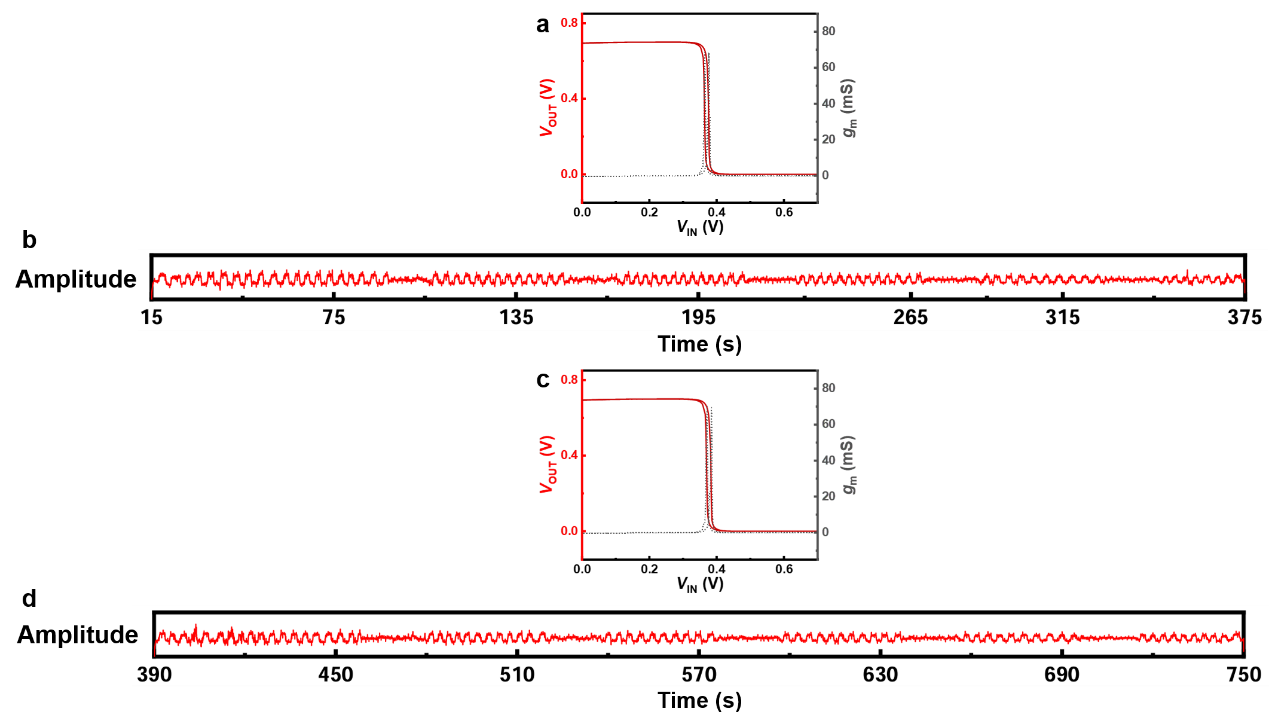


**Figure S4.** EOG monitoring: a) Vm acquisition during the first inverter self-check (15 s). b) EOG monitoring after the first self-check. c) Vm acquisition during the second inverter self-check (15 s). d) EOG monitoring after the second self-check. All EOG signals were processed with a 0.05–30 Hz bandpass filter and baseline correction. Measurements were performed using a PEG-LiCl solid inverter with an Ag side gate under a *V*_DD_ of 0.7 V.


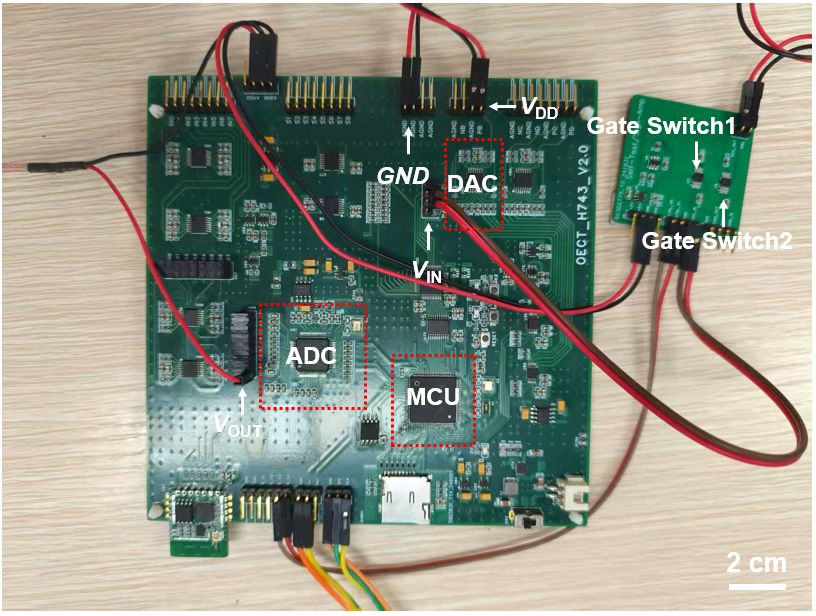


**Figure S5.** Optical image of the dynamic gain optimization system.

**Structure and working principle of dynamic gain modulation system:** The system primarily consists of an STM32H743VIT6 microcontroller (MCU) with peripheral circuits, an AD7768 analog-to-digital converter (ADC), a DAC8562 digital-to-analog converter (DAC), two ADG619 single-pole double-throw (SPDT) switches, and an SD card storage circuit. The STM32H743VIT6 controller employs a 32-bit ARM Cortex-M7 core to drive and control devices such as the ADC, DAC, and ADG switches. The AD7768 is a 24-bit resolution sigma-delta ADC chip that converts continuous voltages into discrete digital signals, used here to collect the output voltage (*V*_OUT_) from the device's *V*_OUT_ terminal. The DAC8562 is a 16-bit resolution serial DAC with ±4 LSB relative accuracy and dual-channel output. It mainly outputs continuous voltage signals corresponding to set values: channel PA supplies continuous voltage and the device's maximum gain (gain__max_) point voltage to the input voltage (*V*_IN_) terminal, while channel PB provides a fixed voltage to the supply voltage (*V*_DD_) terminal. The ADG619 is a CMOS SPDT analog switch that enables signal path switching by configuring its control pin levels. Two ADG619 switches are used for time-division multiplexing of the DAC PA channel output voltage, allowing it to flow through different paths at distinct times.

Upon system power-on reset, initialization parameter configuration is performed for modules including the system clock (CLK), general-purpose input/output ports (GPIO), interrupt ports (INT), serial peripheral interface (SPI), and file system (FATFS). Leveraging the initialized hardware interfaces, subsequent initialization of system peripherals such as the AD7768, DAC8562, and SD card is completed, finalizing the system parameter configuration and peripheral initialization process.

After initialization, the DAC8562 PB channel outputs a fixed voltage of 600 mV to the device's *V*_DD_ terminal, followed by a self-check process to obtain the operating voltage at the device's gain__max_. Specifically:

1. Configure the two ADG619 control pins to a high level, enabling the DAC8562 PA channel voltage to be directly input to the inverter's *V*_IN_ terminal.
2. The PA channel sequentially outputs voltages from 300 to 600 mV (with a 1 mV step) to the device's *V*_IN_ terminal, delaying 50 ms each time before collecting and storing the voltage at the device's *V*_OUT_ terminal via the AD7768.
3. Using the input voltage at the *V*_IN_ terminal as the abscissa and the corresponding collected voltage at the *V*_OUT_ terminal as the ordinate, calculate the point with the maximum slope of the *V*_OUT_ curve. The *V*_IN_ corresponding to this point is the operating voltage at the device's gain__max_.

The time required to obtain the gain__max_ point operating voltage is approximately 50 ms × (600 – 300) = 15000 ms = 15 s, which varies with the scanning voltage range of the PA channel (larger ranges lead to longer times).

After acquiring the operating voltage at the device's gain__max_, human physiological signal collection begins as follows:

1. Configure the two ADG619 control pins to a low level, allowing the DAC8562 PA channel voltage to pass through the human body before transmitting to the inverter's *V*_IN_ terminal.
2. The DAC8562 PA channel fixedly outputs the device's gain__max_ operating voltage, which flows through the human body and inputs to the device's *V*_IN_ terminal.
3. Continuously read the voltage collected by the AD7768 at the device's *V*_OUT_ terminal (data acquisition rate: 4000 Hz), store it in a buffer, and write the data to the SD card when the buffer is full. Continue until 30 s of data are collected to end the continuous acquisition task.

The human physiological signal collection takes approximately 30 s, determined by the number of collected data points (4000 data points equal 1 s based on the AD7768 sampling rate).

Upon completion of human physiological signal collection, a test cycle finishes, and subsequent cycles repeat the self-check process and physiological signal collection. The current test cycle time is 45 s, which can be adjusted by modifying the DAC PA channel output voltage range in the self-check process and the number of data points collected for human physiological signals.

**Table S1.** Signal-to-noise ratio (SNR) of OECT-based complementary inverters for simulated ECG monitoring without or with a modulating system (measured every 10 mins for 90 mins).

| **Testing time (mins)** | **SNR without system (dB)** | **SNR with system (dB)** |
| --- | --- | --- |
| 1 | 28.98±2.43 | 28.98±2.09 |
| 10 | 15.01±1.42 | 28.75±1.87 |
| 20 | 14.72±1.23 | 28.02±1.77 |
| 30 | 14.05±1.02 | 28.25±2.07 |
| 40 | 13.92±1.19 | 28.73±1.60 |
| 50 | 13.71±1.31 | 28.49±2.31 |
| 60 | 13.48±1.08 | 28.59±2.38 |
| 70 | 13.23±1.14 | 29.09±2.33 |
| 80 | 12.65±0.97 | 29.28±2.18 |
| 90 | 12.32±1.13 | 29.15±1.91 |

The error values come from five devices.


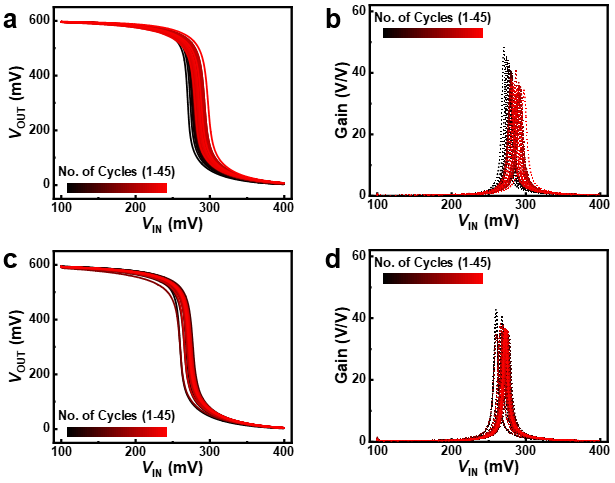


**Figure S6.** Inverter characteristic responses to simulated ECG signals: a) VTC and b) gain of the inverter without the dynamic gain modulating system; c) VTC and d) gain of the inverter with the dynamic gain modulating system.


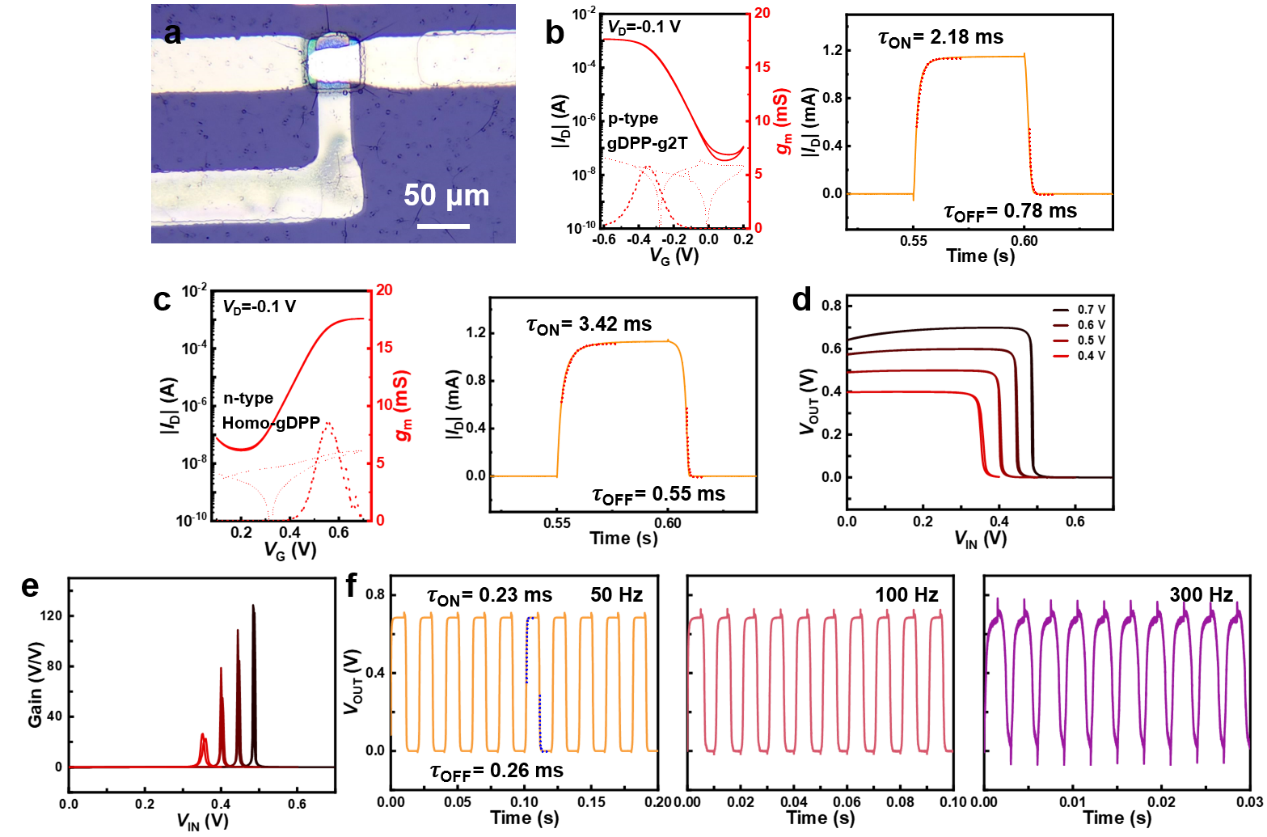


**Figure** **S7.** Device structure, electrical performance, and real-time human ECG monitoring of optimized inverter: a) Optical microscope image. b,c) p/n-type OECT performances. d) VTC, e) voltage gain, and f) transient responses under different frequencies of the inverter. The dimensions of the patterned semiconductor and the encapsulation opening are both 40 × 40 μm² while the dimension of the channel is 30 × 30 μm².


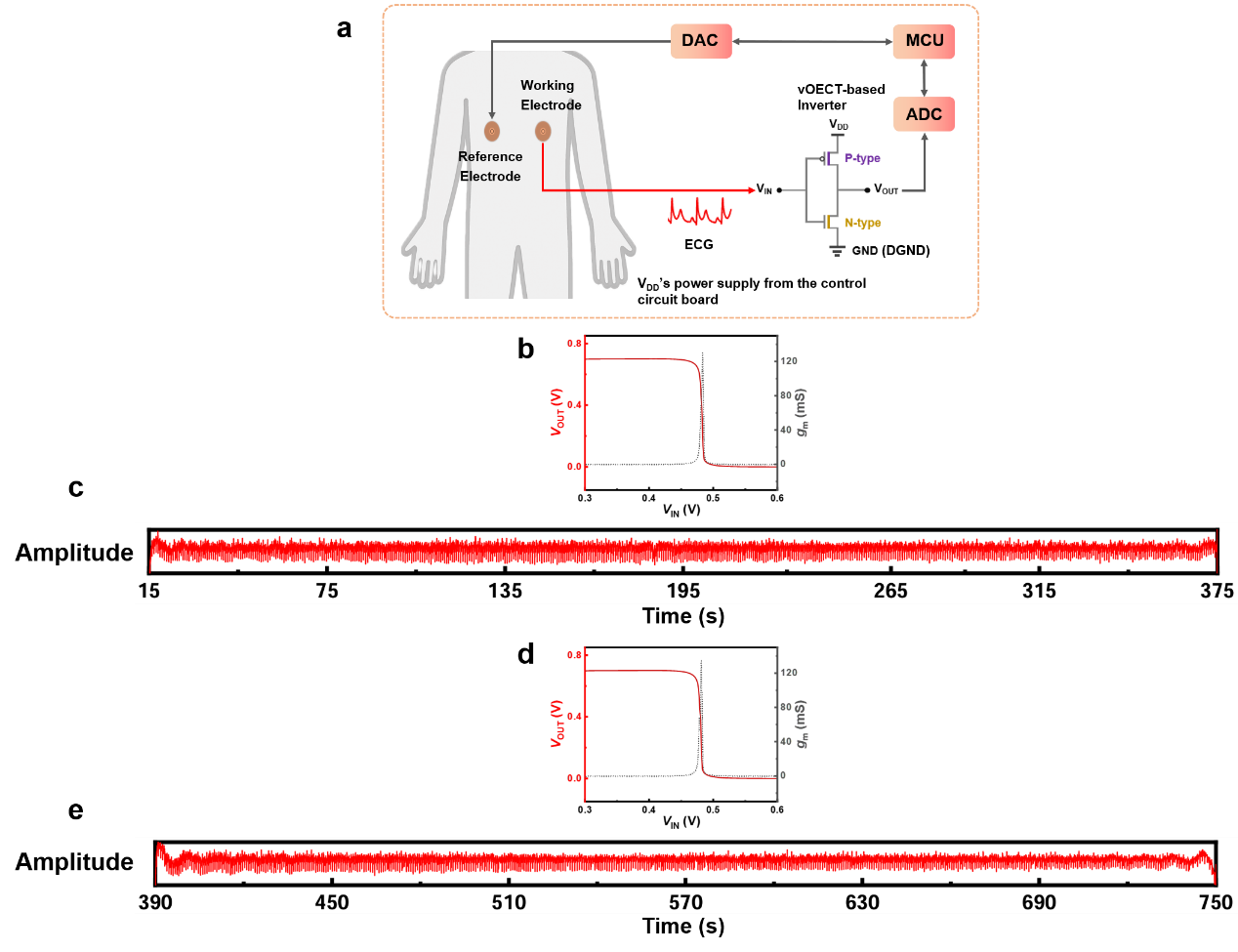


**Figure S8.** Real-time ECG monitoring: a) Schematic of the OECT-based inverter for real-time human ECG detection. b) Vm during the initial 15 s inverter self-check. c) ECG signal after the first self-check. d) Vm during the second 15 s self-check. e) ECG signal following the second self-check. Measurements were conducted with a *V*_DD_ of 0.7 V using PBS electrolyte and an Ag/AgCl floating gate.

**Table S2.** SNR of the OECT-based complementary inverters for real-time human ECG monitoring without or with a modulating system (measured every 10 mins for 90 mins).

| **Testing time (mins)** | **SNR without system (dB)** | **SNR with system (dB)** |
| --- | --- | --- |
| 1 | 27.15±2.61 | 27.63±2.03 |
| 10 | 21.88±2.09 | 28.01±2.18 |
| 20 | 14.60±1.73 | 28.10±2.99 |
| 30 | 13.05±1.68 | 28.13±3.37 |
| 40 | 12.29±1.39 | 27.64±3.22 |
| 50 | 11.65±1.47 | 27.69±3.15 |
| 60 | 10.38±1.55 | 28.09±3.31 |
| 70 | 9.82±1.29 | 28.12±2.76 |
| 80 | 9.43±1.33 | 27.65±3.14 |
| 90 | 8.79±1.23 | 27.32±2.93 |

The error values come from five devices.

**Table S3.** SNR of the OECT-based complementary inverters for EOG monitoring without or with a modulating system (measured every 10 mins for 90 mins).

| **Testing time (mins)** | **SNR without system (dB)** | **SNR with system (dB)** |
| --- | --- | --- |
| 1 | 27.53±2.89 | 27.87±1.87 |
| 10 | 22.01±2.33 | 29.10±2.73 |
| 20 | 15.32±1.89 | 28.03±3.04 |
| 30 | 14.05±1.75 | 29.35±3.41 |
| 40 | 13.32±1.23 | 28.36±3.16 |
| 50 | 12.91±2.01 | 27.79±3.28 |
| 60 | 11.88±1.76 | 29.07±3.47 |
| 70 | 11.53±1.39 | 29.08±2.08 |
| 80 | 11.02±1.52 | 28.32±3.02 |
| 90 | 10.58±1.39 | 28.03±3.13 |

The error values come from five devices.


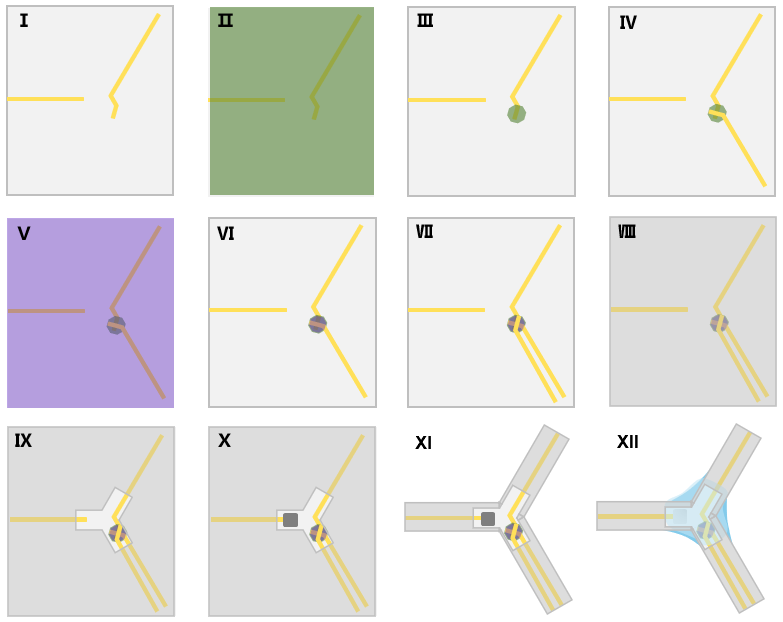


**Figure S9.** Fabrication process of the stretchable OECT-based inverter. Ⅰ) Bottom electrode deposition, Ⅱ) P-type semiconductor (gDPP-g2T) deposition, Ⅲ) P-type channel patterning, Ⅳ) Middle electrode deposition, Ⅴ) N-type semiconductor (Homo-gDPP) deposition, Ⅵ) N-type channel patterning, Ⅶ) Top electrode deposition, Ⅷ) Photoresist (SU8-2002) deposition, Ⅸ) Photoresist development, Ⅹ) Ag gate electrode fabrication, XI) Substrate (PET) patterning, XII) Electrolyte application.

Note, we employ laser etching for BBL semiconductor film patterning; however, the mismatch in thermal expansion coefficients between the Au (14.2 × 10⁻⁶/K) and PET (~60–100 × 10⁻⁶/K) leads to electrode expansion and fracture during laser processing. Therefore, Homo-gDPP/gDPP-g2T inverter, instead of BBL/gDPP-g2T, was fabricated on PET substrates for *in-situ* electrophysiological monitoring, which utilized conventional photo-patterning process for active layer patternning.


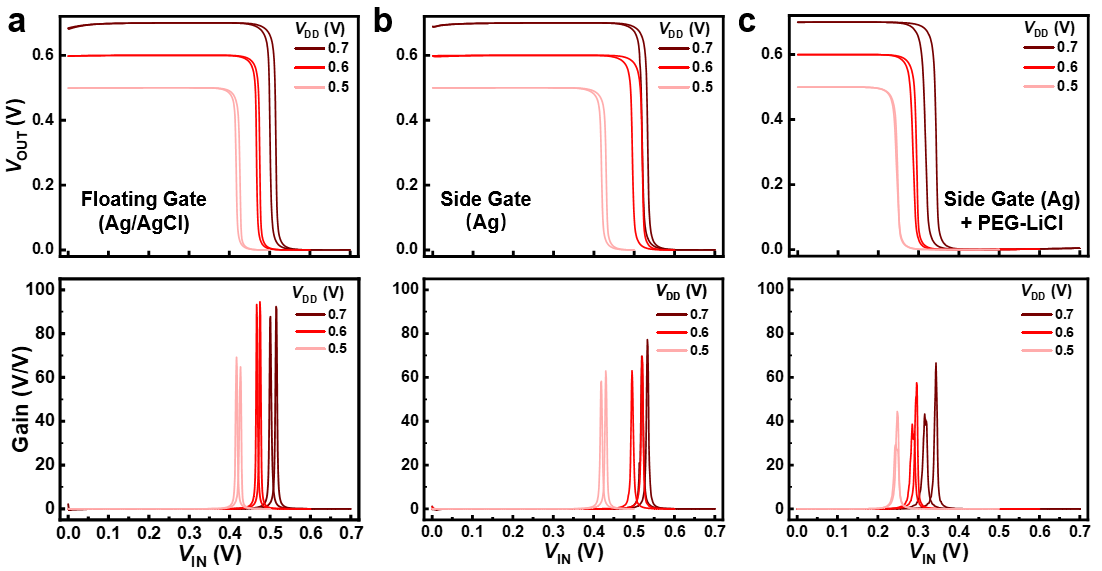


**Figure S10.** VTC and corresponding voltage gains at *V*_DD_=0.5, 0.6, 0.7 V for: a) Ag/AgCl floating-gate inverter; b) Ag side-gate inverter; c) Ag side-gate inverter with quasi-solid-state electrolyte (PEG-LiCl).

The incorporation of stretchability is essential for maintaining stable electrode–skin contact during EOG measurements, especially under ocular and eyelid movements that induce skin strain. Based on Misu et al. (2023), typical periorbital strains remain below 15% (Skin deformation caused by eye and eyelid movements).^[1]^ The 24% stretch margin was selected to exceed this range with a sufficient safety factor, thereby safeguarding signal integrity against motion artifacts.


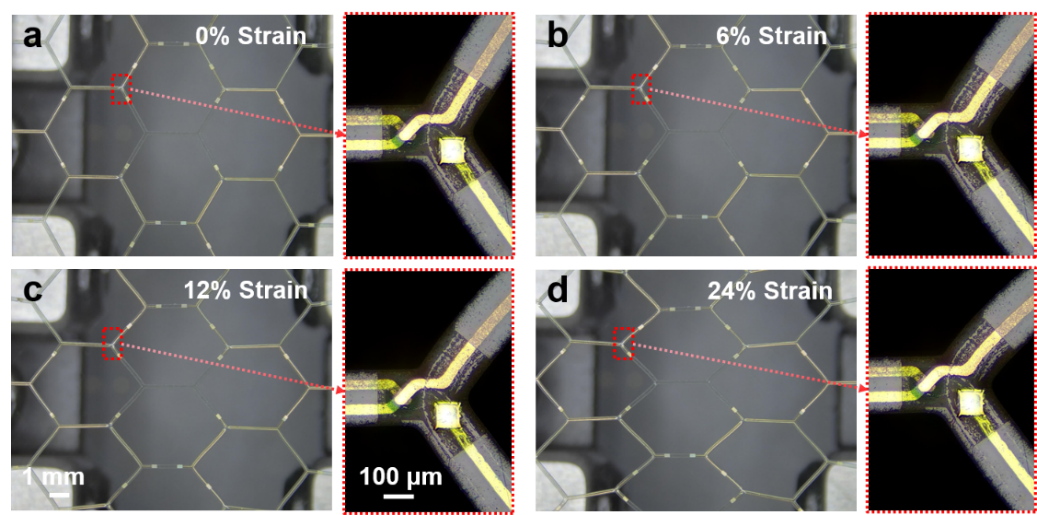


**Figure S11.** Optical microscopy images of the OECT-based stretchable inverter at various tensile strains: a) 0%, b) 6%, c) 12%, and d) 24%.


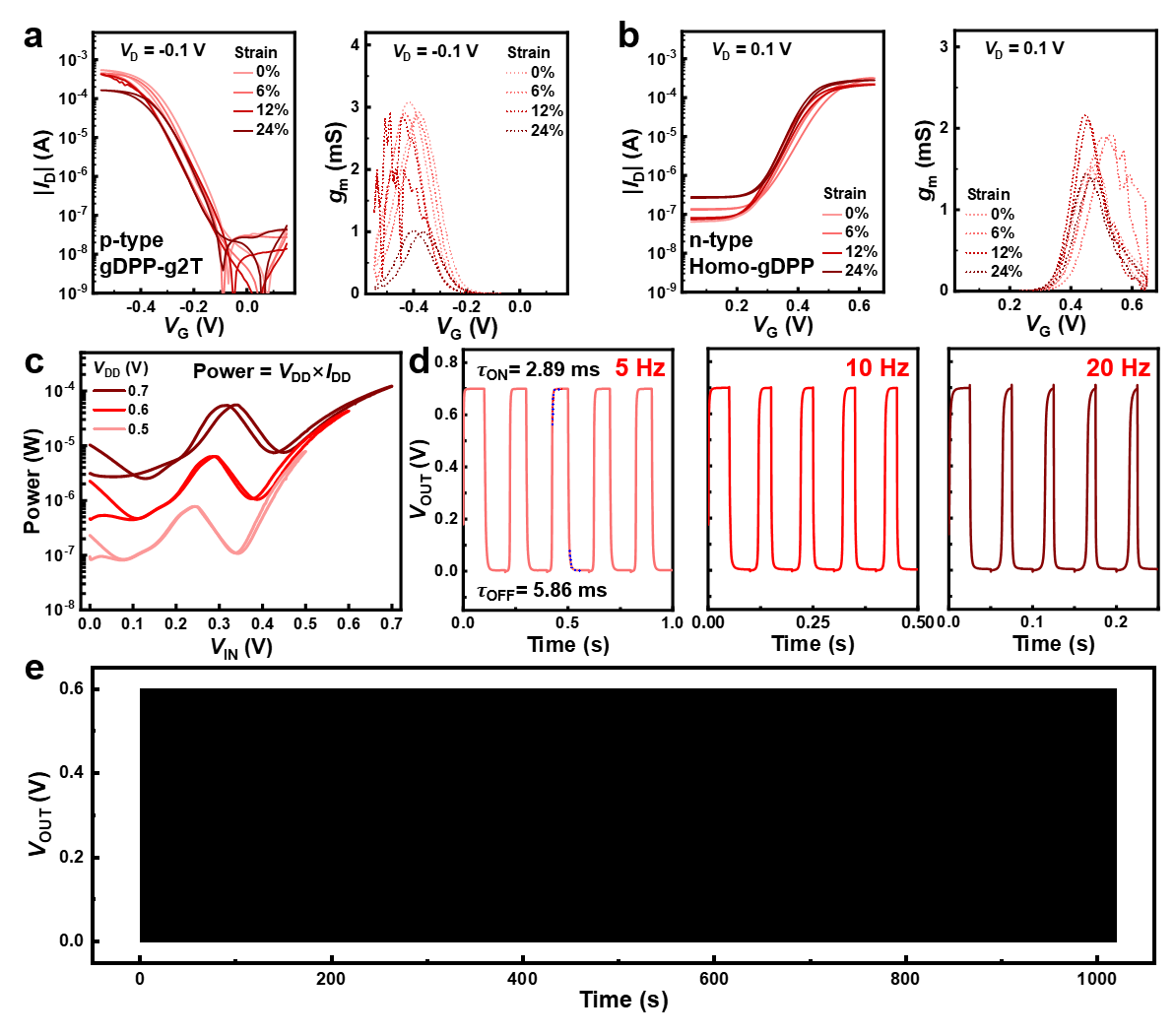


**Figure S12.** Mechanical durability testing of the OECT-based stretchable inverters: a,b) Electrical performance of p/n-type OECT in inverters under 0–24% strain; c–e) Inverter functional testing under 24% strain, c) power consumption, b) transient and frequency response, e) operational stability. All fabricated devices featured uniform channel dimensions of 30 × 30 μm^2^


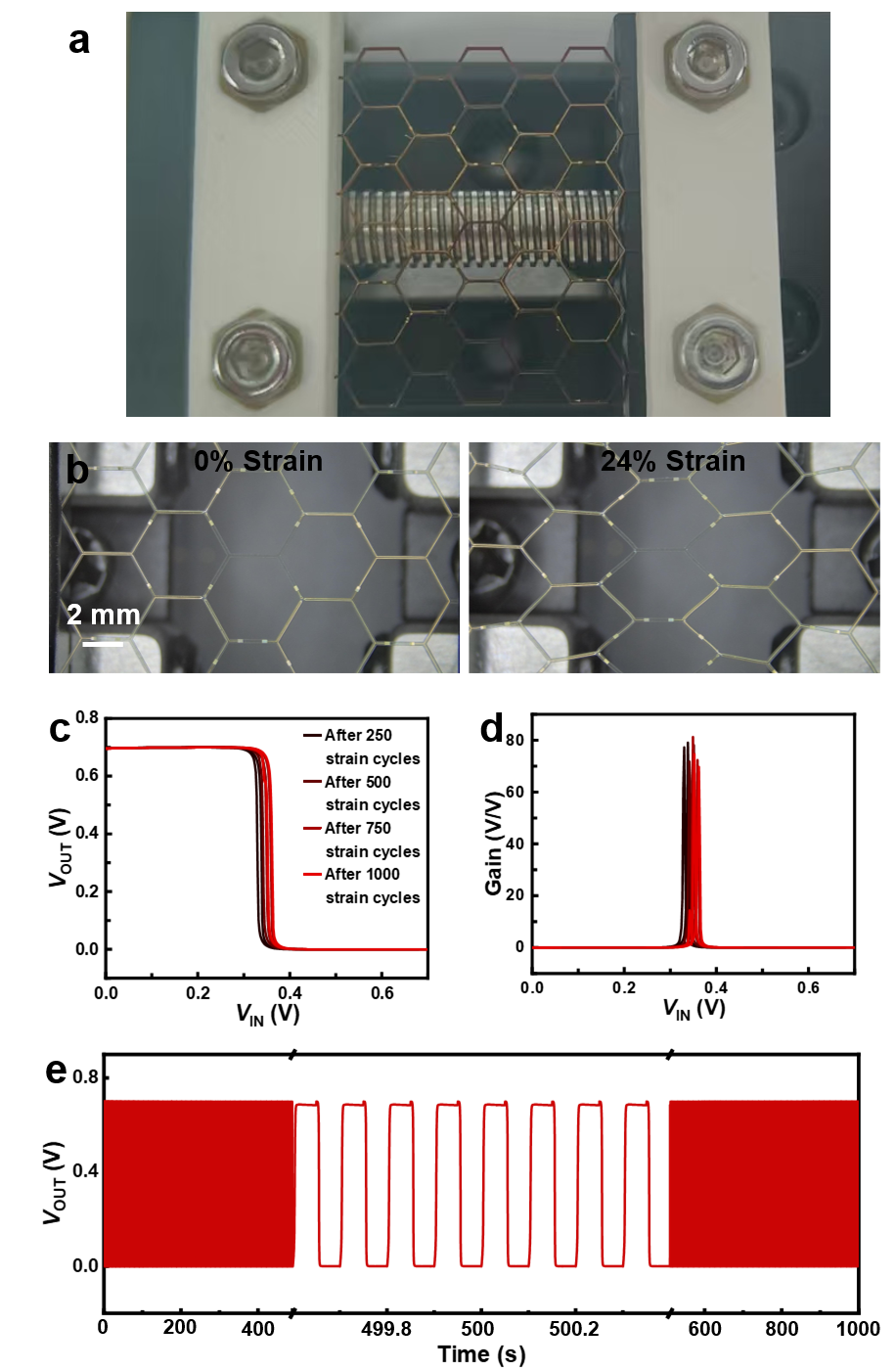


**Figure S13.** Dynamic stretching stability of the inverter: a,b) Optical micrographs under 0% and 24% strain. c–e) Electrical performance: c) VTC, d) voltage gain, and e) cycling stability with an enlarged view of cycles 4996–5004. During this process, 1000 dynamic stretching cycles are applied in 1000 seconds while switching cycle stability of the inverter is actively conducted with a 10 Hz square wave V_IN_; after every 2500 switching cycles, a VTC test is conducted. All measurements were performed at *V*_DD_ = 0.7 V using a PEG-LiCl solid inverter with an Ag side gate.


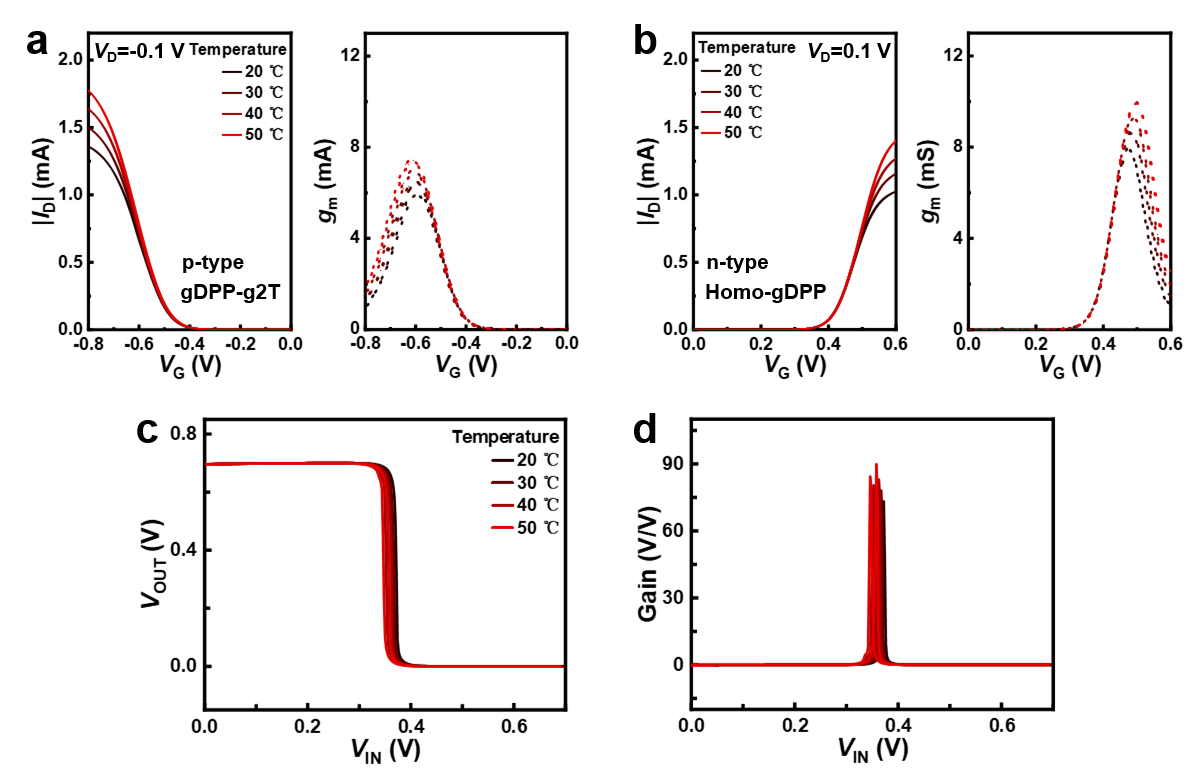


**Figure S14.** Thermal stability characterisation of PEG-LiCl solid inverter with Ag side gate: a, b) p/n-type OECT performance at 20, 30, 40, and 50 °C. c,d) VTC and gain of the inverter across the same temperature range. All measurements were performed under a *V*_DD_ of 0.7 V.


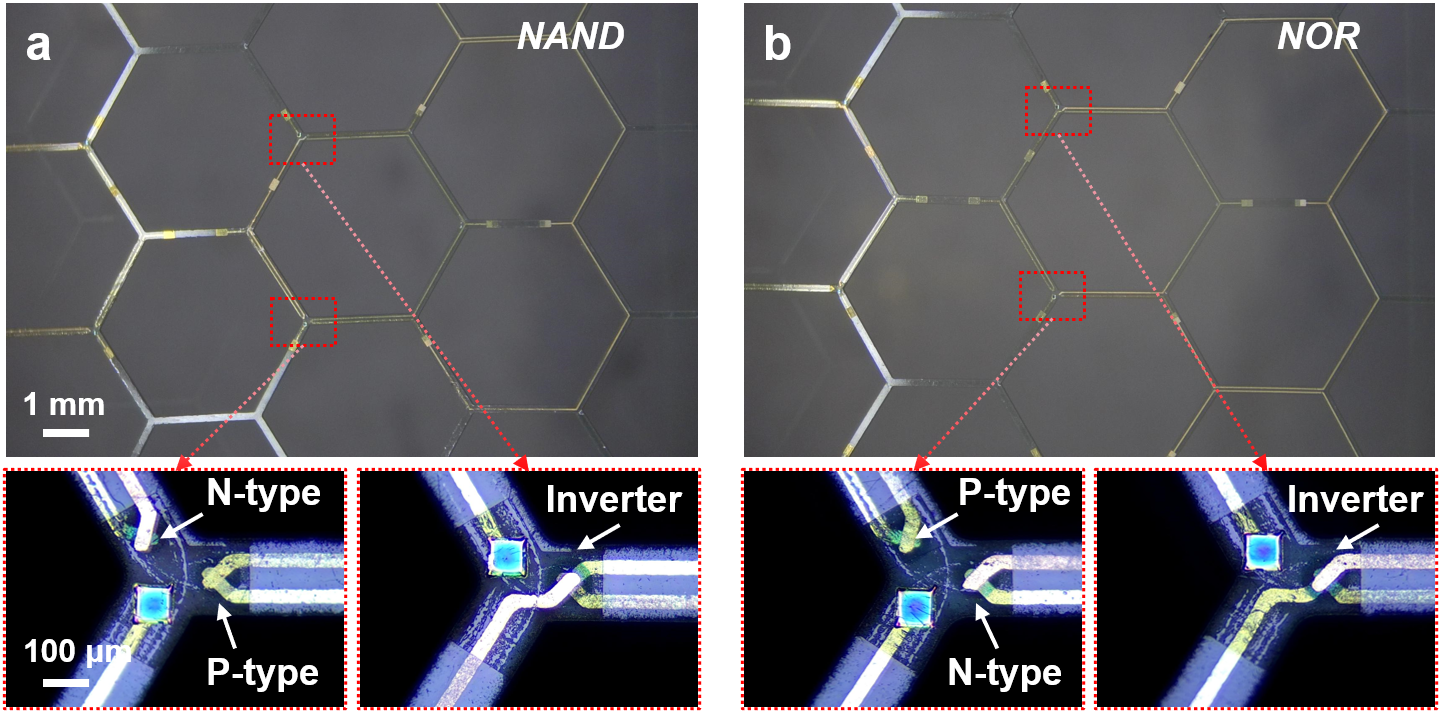


**Figure S15.** a,b) Microscope images of the fabricated stretchable NAND and NOR gates


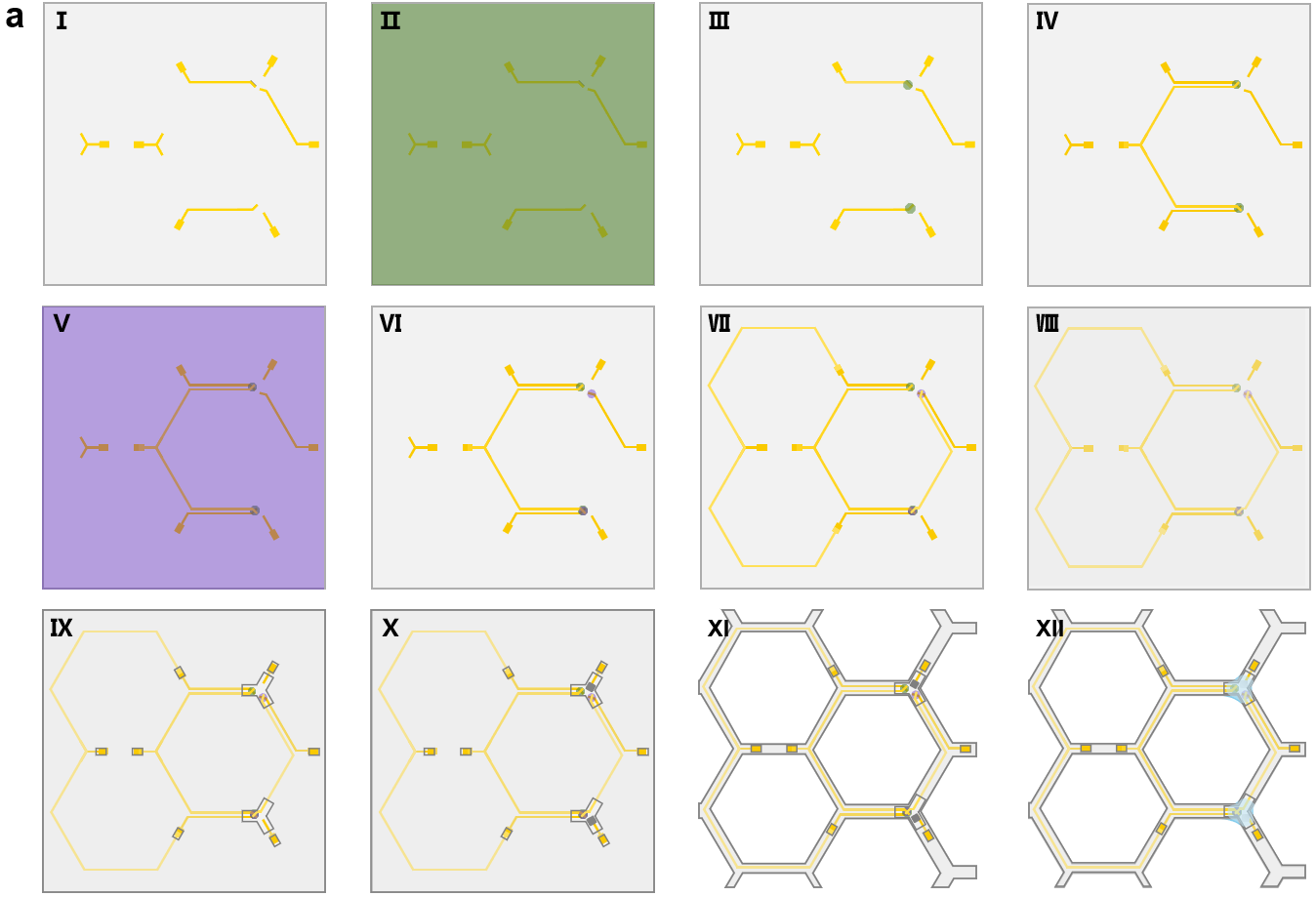


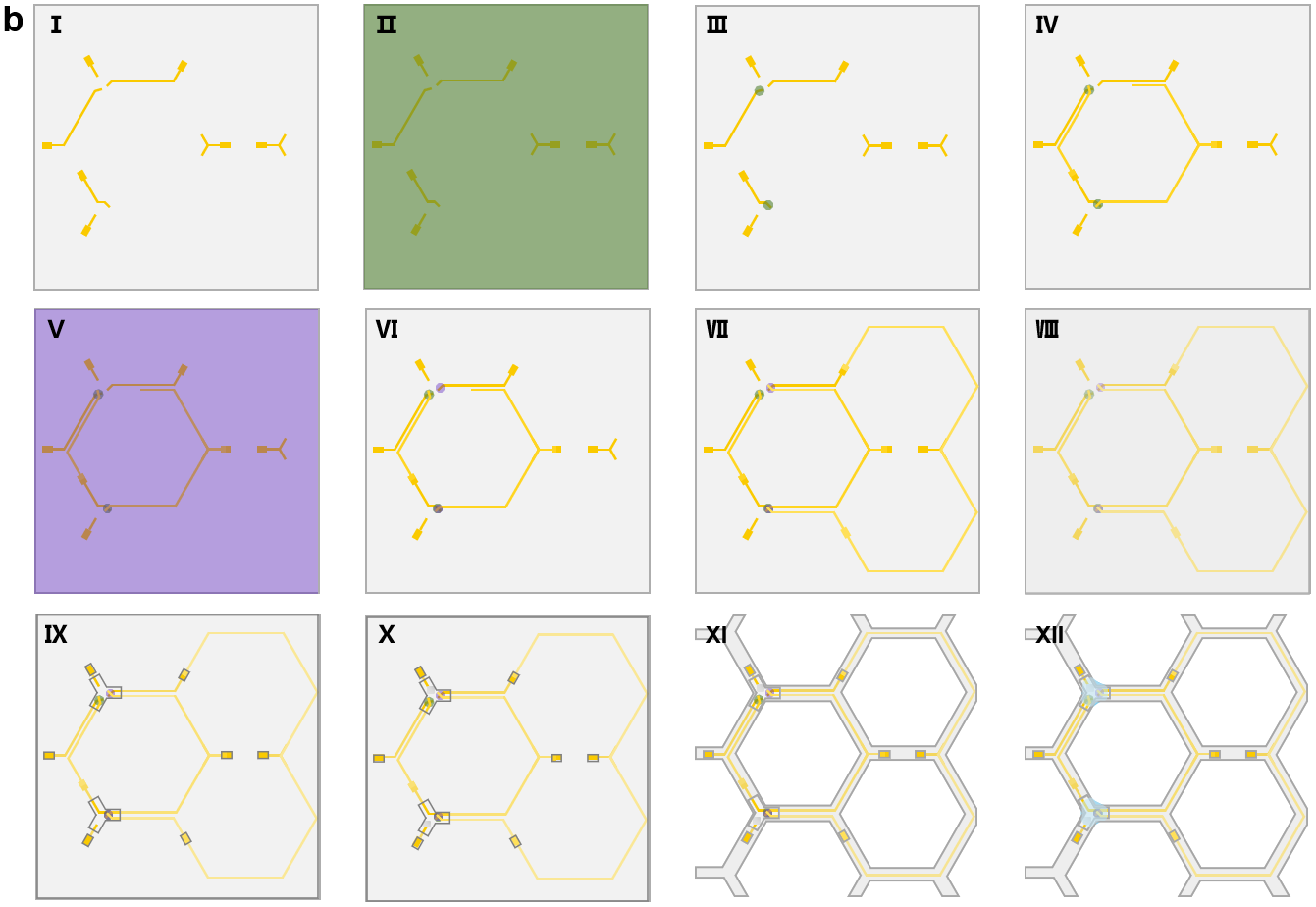


**Figure S16.** Fabrication process of the stretchable a) NAND and b) NOR gates. Ⅰ) Bottom electrode deposition, Ⅱ) P-type semiconductor (gDPP-g2T) deposition, Ⅲ) P-type channel patterning, Ⅳ) Middle electrode deposition, Ⅴ) N-type semiconductor (Homo-gDPP) deposition, Ⅵ) N-type channel patterning, Ⅶ) Top electrode deposition, Ⅷ) Photoresist (SU8-2002) deposition, Ⅸ) Photoresist development, Ⅹ) Ag gate electrode fabrication, XI) Substrate (PET) patterning, XII) Electrolyte application.


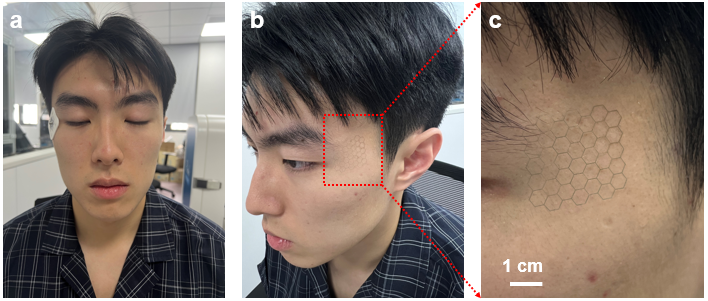


**Figure S17.** a,b) Demonstration of the OECT-based stretchable inverter adhered on the left side of the eye side for *in-suit* EOG monitoring, the reference electrode patch is located on the right side of the human eye side. c) Local enlargement of the inverter.

**Table S4.** SNR of OECT-based stretchable complementary inverters for *in-situ* EOG monitoring without and with a modulating system (measured every 10 mins for 90 mins).

| **Testing time (mins)** | **SNR without system (dB)** | **SNR with system (dB)** |
| --- | --- | --- |
| 1 | 32.93±4.51 | 33.18±2.53 |
| 10 | 20.32±1.89 | 33.12±2.65 |
| 20 | 16.06±1.76 | 32.99±2.71 |
| 30 | 14.18±1.38 | 32.91±3.75 |
| 40 | 13.89±1.37 | 32.84±3.13 |
| 50 | 13.65±1.24 | 32.95±2.58 |
| 60 | 13.34±2.03 | 32.79±2.98 |
| 70 | 12.87±1.29 | 32.76±3.41 |
| 80 | 12.53±1.07 | 32.53±3.86 |
| 90 | 12.21±1.28 | 32.59±3.03 |

The error values come from five devices.


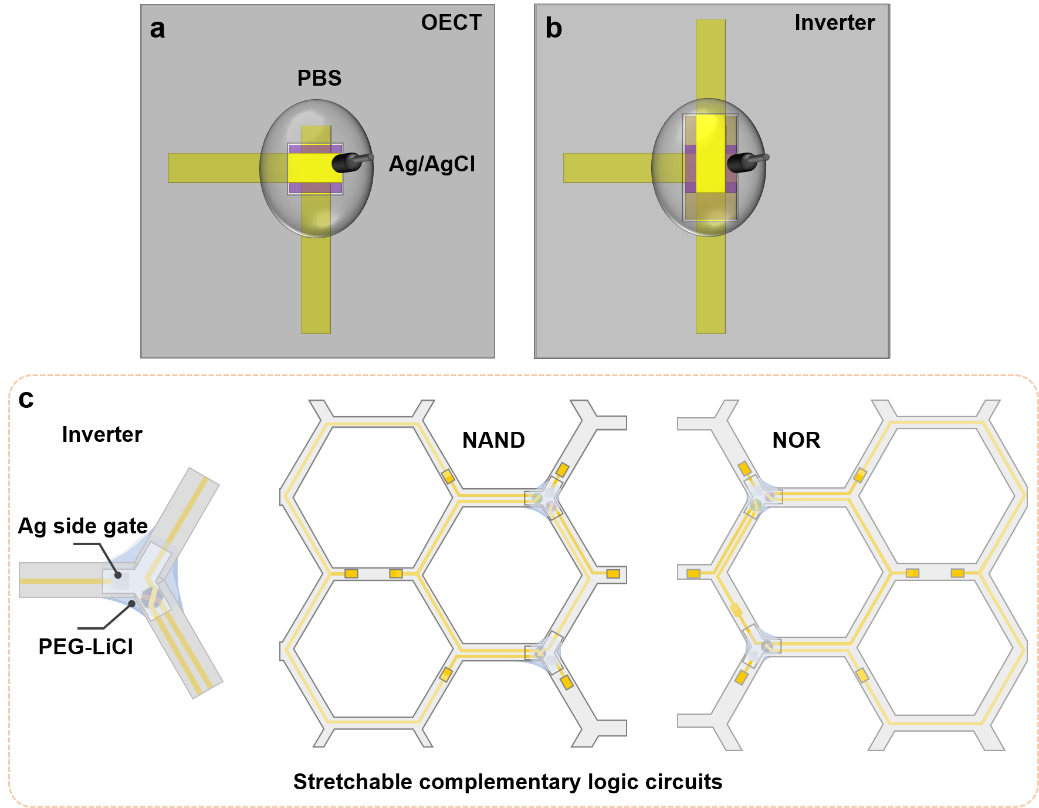


**Figure S18.** a,b) Schematic illustrations of the fabricated OECT and inverter structures (on glass substrate). c) Schematic of the fabricated stretchable logic circuit (on PET substrate).

**Reference**

[1] T. Misu, H. Ishihara, S. Nagashima, Y. Doi, A. Nakatani, Visualization and Analysis of Skin Strain Distribution in Various Human Facial Actions, *Mech. Eng. J.* **2023**, *10*, 23.
